# Changes in physical activity, sedentary behaviour and sleep following pulmonary rehabilitation: a systematic review and network meta-analysis

**DOI:** 10.1183/16000617.0225-2023

**Published:** 2024-04-10

**Authors:** James Manifield, Yousuf Chaudhry, Sally J. Singh, Thomas J.C. Ward, Maxine E. Whelan, Mark W. Orme

**Affiliations:** 1 Department of Respiratory Sciences, University of Leicester, Leicester, UK; 2 Centre for Exercise and Rehabilitation Science, University Hospitals of Leicester NHS Trust, National Institute for Health and Care Research (NIHR) Leicester Biomedical Research Centre (BRC) – Respiratory, Leicester, UK; 3 Centre for Healthcare and Communities, Coventry University, Coventry, UK

## Abstract

**Background:**

The variety of innovations to traditional centre-based pulmonary rehabilitation (CBPR), including different modes of delivery and adjuncts, are likely to lead to differential responses in physical activity, sedentary behaviour and sleep.

**Objectives:**

To examine the relative effectiveness of different pulmonary rehabilitation-based interventions on physical activity, sedentary behaviour and sleep.

**Methods:**

Randomised trials in chronic respiratory disease involving pulmonary rehabilitation-based interventions were systematically searched for. Network meta-analyses compared interventions for changes in physical activity, sedentary behaviour and sleep in COPD.

**Results:**

46 studies were included, and analyses were performed on most common outcomes: steps per day (k=24), time spent in moderate-to-vigorous physical activity (MVPA; k=12) and sedentary time (k=8). There were insufficient data on sleep outcomes (k=3). CBPR resulted in greater steps per day and MVPA and reduced sedentary time compared to usual care. CBPR+physical activity promotion resulted in greater increases in steps per day compared to both usual care and CBPR, with greater increases in MVPA and reductions in sedentary time compared to usual care, but not CBPR. Home-based pulmonary rehabilitation resulted in greater increases in steps per day and decreases in sedentary time compared to usual care. Compared to usual care, CBPR+physical activity promotion was the only intervention where the lower 95% confidence interval for steps per day surpassed the minimal important difference. No pulmonary rehabilitation-related intervention resulted in greater increases in MVPA or reductions in sedentary time compared to CBPR.

**Conclusion:**

The addition of physical activity promotion to pulmonary rehabilitation improves volume of physical activity, but not intensity, compared to CBPR. High risk of bias and low certainty of evidence suggests that these results should be viewed with caution.

## Shareable abstract

**CBPR interventions increased both the volume (steps per day) and the intensity (MVPA) of PA, and reduced sedentary time compared with usual care (no PR). The addition of PA promotion adjuncts to PR further increased changes in PA volume, but not intensity.** https://bit.ly/42DHjav

## Introduction

Physical activity, sedentary behaviour and sleep make up an individual's 24-h day, and can be considered “24-h movement behaviours” which are independently and synergistically important for our health. Physiologically, physical activity is any bodily movement produced by skeletal muscles resulting in energy expenditure [1]. People living with chronic respiratory diseases (CRDs) are not only less physically active than healthy adults [2], but also less physically active when compared with people living with a range of other noncommunicable diseases [3]. In COPD, low physical activity is associated with an increased risk of hospitalisation [4] and premature death [5]. Sedentary behaviour, defined as any waking behaviour of an energy expenditure ≤1.5 metabolic equivalents, while in a sitting, reclining or lying posture [6], has also been associated with premature mortality [7]. Poor sleep quality [8], sleep dissatisfaction [9] and inadequate sleep [10] are common features of CRDs, linked to more severe symptoms and a greater risk of experiencing an acute exacerbation [11].

While distinct behaviours with independent health risks, physical activity, sedentary behaviour and sleep are intrinsically interrelated behaviours. The interplay between physical activity, sedentary behaviour and sleep affects health [12, 13], reflected within the most recent World Health Organization and Canadian 24-h movement guidelines [14, 15]. The 24-h movement profile has seldom been examined in the context of CRDs and pulmonary rehabilitation [16, 17]. Given the low levels of physical activity, high sedentary behaviour and poor sleep quality in CRD populations, interventions, such as pulmonary rehabilitation, that target behaviour change, require evaluation from a 24-h perspective.

Pulmonary rehabilitation is a highly effective and cornerstone intervention for people living with CRDs to improve outcomes of exercise capacity, health-related quality of life and breathlessness [18, 19]. The American Thoracic Society/European Respiratory Society 2013 definition of pulmonary rehabilitation, which includes “behaviour change, … to promote the long-term adherence to health-enhancing behaviours” [20], demonstrates the fundamental role of physical activity and the critical role of health behaviour change [21]. Narrative reviews examining changes in physical activity following pulmonary rehabilitation have shown inconsistent findings between studies, with some reporting an increase and others failing to show statistical or meaningful changes [22–24]. Therefore, the translation of improved exercise capacity leading to increased physical activity following pulmonary rehabilitation cannot be presumed [24].

The emergence of newer models of pulmonary rehabilitation, such as home-based pulmonary rehabilitation (HBPR) [25–28], have offered complementary person-centred options (and patient choice) for services to improve access, uptake and completion [21]. The range of pulmonary rehabilitation models now available have led to innovations and adjuncts in pulmonary rehabilitation components including health behaviour change [21]. The variety of available pulmonary rehabilitation-based interventions and associated adjuncts are likely to lead to differential responses in movement behaviours, but this has been largely unexplored. physical activity interventions have shown promise in increasing physical activity in CRDs [29], but alone are not a substitute for pulmonary rehabilitation programmes, with a mixture of strategies likely needed to elicit behaviour change [21].

Previous pairwise meta-analyses in this area have shown that the addition of physical activity interventions to pulmonary rehabilitation results in significantly greater increases in steps per day in COPD compared to pulmonary rehabilitation alone [30, 31]. The effect of these adjuncts to pulmonary rehabilitation on intensity-related physical activity outcomes (*e.g.* time spent in moderate-to-vigorous physical activity (MVPA)), sedentary behaviour and sleep are yet to be explored.

A network meta-analysis (NMA) is a statistical technique to determine the effectiveness of a range of interventions. This method compares multiple treatments by incorporating both direct and indirect evidence, allowing for a more precise estimate compared to estimates from direct studies alone [32]. NMAs have been conducted previously in the area of pulmonary rehabilitation research [33, 34], but to date have not been utilised for movement behaviour outcomes such as physical activity.

Accordingly, the present review aims to 1) investigate the relative effectiveness of different pulmonary rehabilitation-based interventions of short-term change in 24-h movement behaviours (*i.e.* physical activity, sedentary behaviour and sleep), and 2) investigate the methods used to measure these behaviours in randomised trials relating to pulmonary rehabilitation in people living with CRD.

## Methods

The protocol for this systematic review was prospectively registered on the International Prospective Register of Systematic Reviews (PROSPERO; identifier CRD42022371664), with the type of meta-analysis not pre-specified. This review is reported in accordance with the Preferred Reporting Items for Systematic Reviews and Meta-Analyses (PRISMA) guidelines [35].

### Search strategy

The search strategy was developed by M.W. Orme and M.E. Whelan using appropriate subject headings for the searched databases. Three electronic databases (MEDLINE, CINAHL and PsycINFO) were searched on 27 October 2022 to identify relevant articles. The final search strategies included MEdical Subject Headings and free-text terms relating to the population (*e.g.* “chronic obstructive pulmonary disease” and “lung disease”), the intervention (*e.g.* “pulmonary rehabilitation” and “prescribed exercise”) and the outcomes (*e.g.* “physical activit*” and “sedentary*”). The search strategy was adapted for each database. Full search strategies are provided in supplementary material A. Reference lists of included studies were hand searched for additional, potentially eligible articles. There were no restrictions relating to language, sample size or publication date.

### Inclusion and exclusion criteria

Eligible studies were included if they fulfilled the pre-determined Population, Intervention, Comparison, Outcomes and Study criteria, as follows. 1) Population/participants: adults (aged ≥18 years) living with CRD. 2) Interventions/exposures: participants who were enrolled on some form of pulmonary rehabilitation (with or without adjuncts) including both exercise and education components for ≥3 weeks. 3) Comparison or control groups: participants not receiving pulmonary rehabilitation (usual care), participants receiving a different pulmonary rehabilitation intervention that meets the “interventions/exposure” criteria, or a nonrehabilitation intervention (*e.g.* pedometer only). 4) Outcomes of interest (device-based or self-reported questionnaires): physical activity (*e.g.* daily step count or self-reported time spent walking per day), sedentary behaviour (*e.g.* time spent sitting/stationary) and sleep (*e.g.* sleep duration) assessed immediately pre- and post-intervention. 5) Study design: randomised trials. The settings for these trials could be hospital, community and/or home-based (digital and/or nondigital) modes of pulmonary rehabilitation delivery.

Search results were screened using Rayyan software [36]. After removing duplicates, two reviewers (J. Manifield and Y. Chaudhry) independently screened for eligibility based on the inclusion criteria, initially by title and abstract before full texts were assessed. The reviewers were blinded to each other's decisions, and any disagreements were resolved through consultation with a third reviewer (M.W. Orme).

### Data extraction

Data extraction was performed by two reviewers (J. Manifield and M.W. Orme) for each eligible study using a pre-determined, standardised Microsoft Excel form. The data extracted included author information (name of first author and year of publication), participant characteristics (sample size, disease condition, age, sex, and lung function), intervention details (type, duration and frequency of pulmonary rehabilitation), outcomes (movement behaviour(s) measured (*e.g.* physical activity) and method of data collection (*e.g.* pedometer)), as well as baseline and post-intervention values (and/or change scores).

### Risk-of-bias assessment

The Cochrane Risk of Bias 2 tool for randomised trials [37] was used to assess the risk of bias within the included studies. Three authors (J. Manifield, M.E. Whelan and M.W. Orme) independently assessed each included study for each primary outcome using this tool and classified studies as having low, high or some concerns across all domains. An overall summary risk-of-bias judgement was derived for each outcome following published guidelines [37, 38], with the overall risk-of-bias judgement for each study determined by the highest risk-of-bias level across the domains.

### Quality-of-evidence assessment

The quality of evidence pertaining to each outcome assessed within the NMA (*i.e.* daily step count, time spent in MVPA and sedentary time) was individually rated by two authors (J. Manifield and M.W. Orme) according to the Grading of Recommendations Assessment, Development, and Evaluations considerations [39]. Footnotes were provided to explain any decisions to downgrade the quality of evidence.

### Quality-of-reporting assessments

The Template for Intervention Description and Replication (TIDieR) checklist [40] was used as a tool to assess the quality of reporting of the intervention in the included studies. Each category of the TIDieR checklist was coded as adequately reported (score=1) or inadequately reported/absent (score=0). Three authors (J. Manifield, T.J.C. Ward and M.W. Orme) independently assessed each included study for each item, providing a total score (out of 12).

In addition, indicators of quality of reporting and questionnaire/device deployment were examined by considering whether key information was provided. For device-based measures, the checklist items were informed by previous guidelines and reports [16, 41, 42].

### Data synthesis

NMAs were conducted within the software MetaInsight [43] for outcomes with sufficient data (daily step count, time spent in MVPA and sedentary time). A frequentist approach [44] was used (produced from the “netmeta” package for R) for continuous variables and using a random-effects model for variations across studies. This provided mean difference scores and 95% confidence intervals for all interventions compared to the reference treatments (usual care and centre-based pulmonary rehabilitation (CBPR)). Usual care and CBPR were chosen as reference treatments, as these were the most commonly used comparisons observed in the included studies.

The NMAs were restricted to studies that included participants with COPD only to fulfil the assumption of population homogeneity. There were insufficient studies in other CRDs to perform separate NMAs for each disease. Inconsistency results (p-values) were obtained using the “netmeta” package showing the agreement between effect estimates obtained from direct and indirect evidence. The inconsistency results are presented in supplementary material B.

Network plots were created within the data visualisation software Flourish (https://flourish.studio/). For the purpose of the NMAs, a pragmatic approach was followed in which some interventions within included studies were considered to be a particular form of pulmonary rehabilitation even if the authors had not explicitly defined them as such. For example, both the addition of angiotensin-converting enzyme inhibition [45] and tiotropium [46] to pulmonary rehabilitation programmes were classified as “CBPR plus medication”. Groupings of certain interventions are listed in table 1 (*e.g.* pulmonary rehabilitation+physical activity counselling and pulmonary rehabilitation+pedometer step targets into pulmonary rehabilitation+physical activity promotion) were undertaken following discussions with all authors with expertise in this area, as well as members of the pulmonary rehabilitation department within University Hospitals of Leicester NHS Trust (Leicester, UK).

**TABLE 1:** Characteristics of included articles, grouped by their reporting physical activity only; physical activity and sedentary behaviour; physical activity and sleep; and physical activity, sedentary behaviour and sleep

**First author, year [reference]**	**Population**	**Sample size**	**Age years**	**Sex or gender (as reported)**	**Lung function FEV_1_ % pred**	**Experimental group** **(NMA grouping)**	**Comparison group(s)** **(NMA grouping)**
**Physical activity only**							
Aldhahir, 2021 [47]	COPD	Total 68 EG 36 CG 32	Total 72.5 EG 75±6 CG 70±9	EG: male 15 (68) female 7 (32) CG: male 13 (59) female 9 (41) (sex)	EG 59±22 CG 52±19	PR+protein supplementation (CBPR+nutrition)	PR+placebo supplementation (CBPR)
Altenburg, 2015 [48]	COPD	Total 155 EG 78 CG77	Total 62 (54–69)	Male/female: Total 102/53 (sex)	Total: 60 (40–75)	PR+PA counselling (CBPR+PA promotion)	Usual care (Usual care) PR (CBPR)
Bentley, 2020 [49]	COPD	Total 30 EG 19 CG 11	Total 67.5 (60.0–70.5) EG 68.0 (63.0–72.0) CG 66.0 (60.0–70.0)	Male/female: Total 13/17 EG 8/11 CG 5/6 (gender)	Not reported	PR+PA promotion (CBPR+PA promotion)	PR (CBPR)
Burtin, 2015 [50]	COPD	Total 80 EG 40 CG 40	EG 66±7 CG 67±8	Male: EG 86% CG 79% (gender)	EG 45±14 CG 46±18	PR+PA counselling (CBPR+PA promotion)	PR (CBPR)
Cameron-Tucker, 2016 [51]	COPD	Total 65 EG 35 CG 30	Total 69±8.6 EG 68±9.9 CG 70±6.8	Female: Total 36 (55) EG 19 (54) CG 17 (57)	Not reported	Telerehabilitation (HBPR)	Usual care (Usual care)
Camillo, 2020 [52]	COPD	Total 44 EG 24 CG 20	Total 62±8 EG 62±8 CG 62±9	Male/female (% male) Total 28/16 (64) EG 17/7 (71) CG 11/9 (55)	Total 50±18 EG 47±16 CG 54±20	PR with downhill walking therapy (CBPR+downhill walking)	PR with conventional walking therapy (CBPR)
Cerdán-de-Las-Heras, 2021 [53]	IPF	Total 29 EG 15 CG 14	EG 70.1±8.8 CG 72.4±7.6	Male: EG 13 (86.6) CG 8 (57.1)	Not reported	Telerehabilitation (HBPR)	Usual care (Usual care)
Cerdán-de-Las-Heras, 2021 [54]	COPD	Total 54 EG 27 CG 27	EG 67.4±10.2 CG 72.5±7.4	Male: EG 16 (51.6) CG 15 (48.4)	EG 36.1±14.1 CG 32.8±8.5	Telerehabilitation (HBPR)	CBPR (CBPR)
Cerdán-de-Las-Heras, 2022 [55]	Sarcoidosis	Total 30 EG 15 CG 15	EG 56.1±14.4 CG 51.6±12.7	Male: EG 10 (66) CG 9 (60)	EG 79.33±16.36 CG 63.00±23.94	Telerehabilitation (HBPR)	Usual care (Usual care)
Chaplin, 2022 [56]	COPD	Total 103 EG 51 CG 52 For baseline characteristics, complete accelerometer data: EG 20 CG 34	EG 68.3±6.5 CG 67.4±8.6	Male/female: EG 18/2 CG 19/15 (gender)	EG 54.2±26.9 CG 55.8±19.4	Web-based PR (HBPR)	Conventional PR (CBPR)
Curtis, 2016 [45]	COPD	Total 78 EG 39 CG 39	EG 66±10 CG 68±7	Female: EG 55% CG 59% (sex)	EG 48.2±22.5 CG 51.6±20.2	PR+ACEi (CBPR+medication)	PR+placebo (CBPR)
de Blok, 2006 [57]	COPD	Total 21 EG 10 CG 11	EG 65.7±10.4 CG 62.5±12.3	Male/female: EG 5/5 CG 4/7 (gender)	EG 52±22 CG 43±13	PR+PA counselling (CBPR+PA promotion)	PR (CBPR)
Duiverman, 2008 [58]	COPD	Total 72 EG 37 CG 35	EG 63±10 CG 61±7	Male/female EG: 18/13 CG: 17/18 (sex)	Not reported	PR+NIPPV (CBPR+NIPPV)	PR (CBPR)
Effing, 2011 [59]	COPD	Total 159 EG 80 CG 79	EG 62.9±8.1 CG 63.9±7.8	Male: EG 58.5% CG 57.9%	EG 49.6±14.2 CG 50.5±17.0	Community-based physiotherapeutic exercise programme (COPE-active) (CBPR)	Self-management (Self-management)
Felcar, 2018 [60]	COPD	Total 70 EG 34 CG 36	EG 69±9 CG 68±8	Male/female: EG 14/6 CG 9/7	EG 48±17 CG 46±14	Water-based exercise training (CBPR water-based)	Land-based exercise training (CBPR)
Gaunaurd, 2014 [61]	IPF	Total 25 EG 14 CG 11	EG 71±6 CG 66±7	Not reported	EG 60±11 CG 61±14	PR (CBPR)	Usual care (Usual care)
Jarosch, 2020 [62]	IPF	Total 54 EG 36 CG 18	EG 68±9 CG 65±10	Male: EG 25 (76) CG 13 (81) (gender)	Not reported	PR (CBPR)	Usual care (Usual care)
José, 2021 [63]	Bronchiectasis	Total 63 EG 33 CG 30	EG 44.42±16.16 CG 49.27±14.10	Female: EG 16 (48.5) CG 18 (60)	EG 55.15±27.19 CG 51.21±0.56	HBPR (HBPR)	Educational booklet (PA promotion)
Kesten, 2008 [46]	COPD	Total 108 EG 55 CG 53	EG 65.9±8.8 CG 67.3±6.9	Male: EG 55% CG 59%	EG 32.6±12.4 CG 36.2±12.2	PR+tiotropium (CBPR+medication)	PR (CBPR)
Nolan, 2017^#^ [64]	COPD	Total 152 EG 76 CG 76	Total 68±9 EG 69±9 CG 68±8	Male: Total 110 (72) EG 56 (74) CG 54 (71) (sex)	Total 50.5±21.2 EG 50.6±20.7 CG 50.3±21.8	PR+pedometer step targets (CBPR+PA promotion)	PR (CBPR)
O’Neill, 2018 [65]	COPD	Total 49 EG 23 CG 26	Total 64.4±8.6 EG 61.1±8.5 CG 67.2±7.8	Male/female: Total 24/25 EG 13/10 CG 11/15 (gender)	Total 56±23 EG 57±24 CG 54±23	PA intervention (PA promotion)	PR (CBPR)
Pavitt, 2020 [66]	COPD	EG 57 CG 65	EG 70 (64–78) CG 68 (62–74)	Female EG 27 (42) CG 26 (41) (gender)	EG 53 (37–65) CG 48 (33–63)	PR+beetroot juice (CBPR+nutrition)	PR+placebo beetroot juice (CBPR)
Perez-Bogerd, 2018 [67]	ILD	Total 60 EG 30 CG 30	EG 64±13 CG 64±8	Men: EG 22 (73) CG 15 (50) (gender)	EG 76±18 CG 77±17	PR (CBPR)	Usual care (Usual care)
Polgar, 2021^#^ [68]	COPD	Total 152 EG 76 CG 76	Total 68±9 EG 69±9 CG 68±8	Male: Total 110 (72) EG 56 (74) CG 54 (71) (sex)	Total 50.5±21.2 EG 50.6±20.7 CG 50.3±21.8	PR+pedometer step targets (CBPR+PA promotion)	PR (CBPR)
Polkey, 2018 [69]	COPD	Total 120 EG 60 CG 60	Not reported	Not reported	EG 48.7±13.4 CG 47.1±15.4	Tai chi (Tai chi)	PR (CBPR)
Selzler, 2021 [70]	COPD	Total 207 EG 108 CG 99	EG 66±8 CG 67±9	Female EG 53% CG 38% (sex)	EG 55±24 CG 56±20	PR+enhanced education (CBPR+enhanced education)	PR (CBPR)
Sewell, 2005 [71]	COPD	Total 180 EG 90 CG 90	EG 67.33±8.41 CG 69.34±8.73	Men/women: EG 51/39 CG 60/30	Not reported	Individually targeted PR programme (CBPR)	General PR programme (General exercise)
Troosters, 2018 [72]	COPD	Total 304 EG 76 CG1 75 CG2 76 CG3 76	EG 64.7±6.5 CG1 64.2±6.5 CG2 65.4±6.3 CG3 64.9±6.9	Male: EG 42 (60.0) CG1 46 (70.8) CG2 51 (76.1) CG3 45 (62.5)	EG 57±13 CG1 56±14 CG2 57±13 CG3 59±11	SMBM+tiotropium/ olodaterol+exercise training (CBPR+medication)	CG1: SMBM+placebo (Self-management) CG2: SMBM+tiotropium (Self-management+medication) CG3: SMBM+tiotropium/olodaterol (Self-management+medication)
van de Bool, 2017 [73]	COPD	Total 81 EG 42 CG 39	EG 62.8±1.3 CG 62.2±1.3	Male: EG 42.9% CG 59.0% (gender)	EG 57.0±3.3 CG 53.0±2.8	PR+nutritional supplementation (CBPR+nutrition)	PR+placebo supplementation (CBPR)
Varas, 2018 [74]	COPD	Total 40 EG 21 CG 19	EG 69.5±7.4 CG 64.8±9.1	Men: EG 18 (85.7) CG 13 (68.4) (gender)	EG 45.8±16.5 CG 52.3±15.7	PR+pedometer feedback (CBPR+PA promotion)	General PA recommendations (PA promotion)
Wallaert, 2020 [75]	Sarcoidosis	Total 38 EG 20 CG 18	EG 57.5 (48.0–63.5) CG 57.5 (49–65)	Male/female: EG 10/10 CG 7/11 (sex)	EG 66.8±20.2 CG 61.1±16.9	PR (CBPR)	PA counselling (PA promotion)
**Physical activity and sedentary behaviour**							
Armstrong, 2021 [76]	COPD	Total 48 EG 24 CG 24	EG 71±9 CG 73±9	Male/female: EG 9/15 CG 9/15 (gender)	EG 51±19 CG 50±17	PR+PA modification (CBPR+PA promotion)	PR (CBPR)
Breyer, 2010 [77]	COPD	Total 65 EG 32 CG 33	Total 60.3±8.5 EG 61.9±8.9 CG 59.0±8.0	Male: Total 45% EG 47% CG 43% (sex)	Total 46.3±17.6 EG 48.1±19.1 CG 47.1±16.3	PR+nordic walking (CBPR)	Usual care (Usual care)
Cox, 2022 [27]	CRD (including COPD, ILD, bronchiectasis, asthma)	Total 142 (100 COPD) EG 71 CG 71	EG 68±9 CG 67±9	Male/female: EG 30/41 CG 36/35	EG 59±25 CG 63±26	Telerehabilitation (HBPR)	CBPR (CBPR)
Cruz, 2016 [78]	COPD	Total 32 EG 16 CG 16	EG 68.8±8.2 CG 64.1±8.2	Male: EG 13 (81.2) CG 14 (87.5) (sex)	EG 65.5±21.1 CG 68.4±19.7	PR+PA focused behavioural intervention (CBPR+PA promotion)	PR (CBPR)
Geidl, 2021 [79]	COPD	Total 327 EG 167 CG 160	Total 58.01±5.43 EG 58.01±5.51 CG 58.03±5.47	Male: Total 69% EG 68.7% CG 69.4%	Total 53.51±18.47 EG 53.05±18.39 CG 54.00±18.61	PR+pedometer-based PA promotion (CBPR+PA promotion)	PR (CBPR)
Hansen, 2020 [80]	COPD	Total 134 EG 67 CG 67	Total 68.3±9.0 EG 68.4±8.7 CG 68.2±9.4	Female: Total 74 (55) EG 35 (52) CG 39 (58) (sex)	Total 33.1±9.4 EG 32.6±10.3 CG 33.7±8.4	Telerehabilitation (HBPR)	CBPR (CBPR)
Holland, 2017^¶^ [28]	COPD	Total 166 EG 80 CG 86	EG 69±13 CG 69±10	Male/female: EG 48/32 CG 51.35	EG 52±19 CG 49±19	HBPR (HBPR)	CBPR (CBPR)
Horton, 2021 [81]	COPD	EG 63 CG 55	EG 67±9.2 CG 67±6.9	Male/female: EG 43/20 CG 37/18	EG 47.22±18.03 CG 51.43±18.77	HBPR (HBPR)	CBPR (CBPR)
Kawagoshi, 2015 [82]	COPD	EG 12 CG 15	EG 74±8 CG 75±9	Male/female: EG 10/2 CG 14/1 (gender)	EG 58.0±23.2 CG 60.6±20.8	PR+pedometer feedback (HBPR+PA promotion)	PR (HBPR)
Lahham, 2020 [83]	COPD	Total 58 EG 29 CG 29	EG 68±9 CG 67±10	Male/female: EG 17/12 CG 17/12	EG 90±8 CG 92±7	HBPR (HBPR)	Usual care (Usual care)
Louvaris, 2016 [84]	COPD	EG 85 CG 43	EG 65±8 CG 67±8	Male/female: EG 68/17 (80/20) CG 36/7 (84/16)	EG 48.8±19.4 CG 44.9±19.0	PR with interval training (CBPR)	Usual care (Usual care)
Park, 2020 [85]	COPD	Total 44 EG 23 CG 21	Total 67.88±10.49 EG 70.45±9.40 CG 65.06±11.12	Male: Total 33 (78.6) EG 19 (86.4) CG 14 (70.0) (gender)	Total 65.02±21.57 EG 61.00±18.73 CG 69.45±24.02	PR+app-based self-management (CBPR+PA promotion)	PR (CBPR)
Rausch Osthoff, 2021 [86]	COPD	Total 43 EG 17 CG 26	EG 70±7 CG 67±9	Male: EG 9 (53) CG 12 (48) (gender)	EG 52.5±20 CG 45.6±16	PR+PA counselling (CBPR+PA promotion)	PR (CBPR)
Vasilopoulou, 2017 [87]	COPD	Total 150 EG 47 CG1 50 CG2 50	EG 66.9±9.6 CG1 66.7±7.3 CG2 64.0±8.0	Men/women: EG 44/3 CG1 38/12 CG2 37/13	EG 49.6±21.9 CG1 51.8±17.3 CG2 51.7±21.0	HBPR (HBPR)	CG1: CBPR (CBPR) CG2: Usual care (Usual care)
**Physical activity and sleep**							
Deering, 2011 [88]	COPD	Total 60 EG 16 CG1 25 CG2 19	EG 65.1±9.7 CG1 67.7±5.3 CG2 68.6±5.5	Men: EG 8 CG1 11 CG2 12	EG 48.8±22.7 CG1 48.5±16.1 CG2 45.8±18.3	PR+acupuncture (CBPR+acupuncture)	CG1: PR (CBPR) CG2: Usual care (Usual care)
**Physical activity, sedentary behaviour and sleep**							
Burge, 2021^¶^ [89]	COPD	Total 73 EG 33 CG 40	EG 65±14 CG 68±10	Female: EG 16 (55) CG 18 (45)	EG 50±20 CG 51±20	HBPR (HBPR)	CBPR (CBPR)
Cedeño de Jesús, 2022 [90]	Noncystic fibrosis bronchiectasis	Total 34 EG 18 CG 16	EG 63±6.14 CG 59.42±9.30	Women: EG 81.25% CG 66.66%	EG 71.88±20.72 CG 74.41±28.44	HBPR (HBPR)	PA promotion (PA promotion)

Data are presented as n, mean±sd, n (%) or median (interquartile range), unless otherwise stated. FEV_1_: forced expiratory volume in 1 s; NMA: network meta-analysis; EG: experimental group; CG: comparison group; PR: pulmonary rehabilitation; CBPR: centre-based pulmonary rehabilitation; PA: physical activity; HBPR: home-based pulmonary rehabilitation; IPF: idiopathic pulmonary fibrosis; ACEi: angiotensin-converting enzyme inhibition; NIPPV: noninvasive positive pressure ventilation; ILD: interstitial lung disease; SMBM: self-management behaviour modification; CRD: chronic respiratory disease. ^#^: data from the same/similar participants; ^¶^: data from the same/similar participants.

When numerical outcome data required for NMAs (*i.e.* within-group mean differences and standard deviations) were not available within included study text or supplementary material, the original study authors were contacted to obtain this information. If it was still not possible to obtain data, the mean±sd of change was estimated from median (interquartile range) values [91] or 95% confidence intervals, or by using the average correlation coefficient for each outcome within included studies reported in considerable detail, in accordance with the Cochrane Handbook [92]. Details for how standard deviations were obtained for studies used within NMAs are provided in the full extraction of results (supplementary material C).

Changes in step count were compared to previously published minimal important difference (MID; 600–1100 steps·day^−1^) following pulmonary rehabilitation in COPD [93]. Sensitivity analyses were performed on the NMAs by using a conservative correlation coefficient estimate (0.5) in place of the calculated correlation coefficient when imputing standard deviation [92] and by removing studies with high risk of bias. Where possible, sensitivity analyses were performed to assess the consistency of findings between studies that reported these outcomes as primary and those that reported them as secondary.

## Results

**FIGURE 1:**
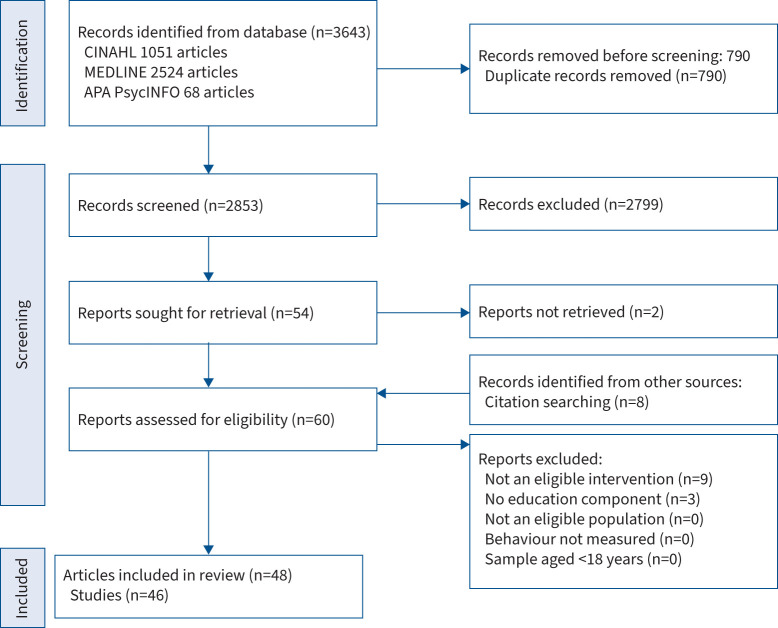
Preferred Reporting Items for Systematic Reviews and Meta-Analyses flow diagram of studies through database search and selection process. APA: American Psychological Association.

The full PRISMA flow diagram of studies through database searching and selection process is shown in figure 1. On completion of full-text screening, 48 articles were considered eligible. Two included articles [68, 89] were secondary analyses of other included studies [21, 64] comprising the same participants. Therefore, participant details from the secondary analyses [68, 89] were excluded when pooling included studies within the present review. These papers [68, 89] were included in the pooling of outcome measure frequencies due to differences in data processing and outcomes with their respective primary articles.

### Characteristics of included subjects

The included studies comprised 4178 participants with a median sample size of 65 (range 21–327) (table 1). Most included articles were conducted in COPD (40 (83.3%) out of 48; n=3806) [27, 28, 45–52, 54, 56–60, 64–66, 68–74, 76–89]. Five studies included interstitial lung disease (n=176) [27, 53, 61, 62, 67], of which three were limited to idiopathic pulmonary fibrosis (n=71) [53, 61, 62]. Three studies included bronchiectasis (n=116) [27, 63, 90]; two included sarcoidosis (n=68) [55, 75]; and one included asthma (n=12) [27].

### Characteristics of interventions

Full details of intervention types are provided in supplementary material D.

Specific interventions used and their interactions between each other are provided as network plots in supplementary material E for all articles, as well as studies reporting changes in daily step count, time spent in MVPA and sedentary time.

The most common interventions across all articles were CBPR (n=39) [27, 28, 45–50, 52, 54, 56–62, 64–71, 73, 75–81, 84–89], HBPR (n=15) [27, 28, 51, 53–56, 63, 80–83, 87, 89, 90] and CBPR+physical activity promotion (n=12) [48–50, 57, 64, 68, 74, 76, 78, 79, 85, 86]. The median duration of pulmonary rehabilitation-based interventions was 8 weeks (range 3 weeks to 1 year). Frequency of CBPR classes ranged from one to six sessions per week, with the average number of sessions per week being three and five for CBPR and HBPR, respectively.

### Methods of 24-h movement behaviour assessment

Of the 4178 included participants, 3436 (COPD n=3088, idiopathic pulmonary fibrosis n=102, sarcoidosis n=61, bronchiectasis n=86 and interstitial lung disease n=60) had data available relating to at least one 24-h movement behaviour following pulmonary rehabilitation. All included articles measured physical activity (k=48, 100%), of which 31 (64.6%) only measured physical activity (table 1). No articles assessed only sedentary behaviour or only sleep. The remaining 17 (35.4%) articles assessed multiple movements behaviours (physical activity+sedentary behaviour (n=14, 29.2%), physical activity+sleep (n=1, 2.1%) or physical activity+sedentary behaviour+sleep (n=2, 4.2%)).

Most articles that included physical activity assessment used device-based methods for data collection (44 out of 48, 91.7%), with the most common devices being the SenseWear Armband (SWA) models (BodyMedia, Pittsburgh, PA, USA) (15 (34.1%) out of 44 or ActiGraph (AG) models (ActiGraph, Pensacola, FL, USA) (14 (31.8%) out of 44. Similar devices were used in articles that assessed sedentary behaviour (SWA and AG, both six out of 16). Articles that assessed physical activity *via* questionnaires most often used the International Physical Activity Questionnaire (three out of nine). All articles that assessed sleep behaviour used device-based measurement (SWA) (three out of three).

### Outcome measures

Across 24-h movement behaviours, 16 different tools were used, generating 66 outcome variables (supplementary material F). The most common outcomes reported within included articles were daily step count (k=38), time spent in MVPA (k=16) and sedentary time (k=11). Of the 48 included articles, only 14 reported a 24-h movement behaviour as a primary outcome, of which, nine out of 14 performed a power calculation for these outcomes (supplementary material G). The full extraction of results of pertaining to physical activity, sedentary behaviour and sleep are reported in supplementary material C.

### NMA: daily step count (steps·day^−1^)

24 studies [28, 45, 47, 48, 50, 52, 54, 56–58, 60, 64, 66, 70, 73, 76, 78–81, 83, 84, 86, 88] (n=1691) were included in the NMA for pre–post changes in average daily step count (steps·day^−1^) in COPD. These consisted of 11 interventions and 55 pairwise comparisons (13 of which were direct data comparisons; supplementary material E). No differences between direct and indirect comparisons were observed (supplementary material B), and all comparisons between interventions are shown in supplementary material H.

**FIGURE 2:**
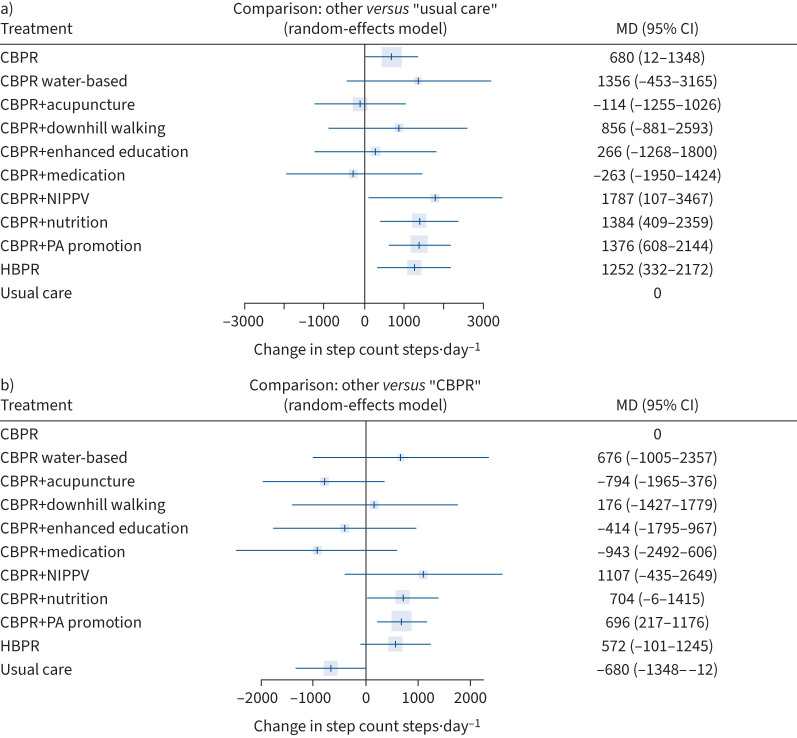
Forest plot comparing change in step count following pulmonary rehabilitation-based interventions for people with COPD with a) usual care and b) centre-based pulmonary rehabilitation (CBPR) (k=24; n=1691). MD: mean difference; NIPPV: noninvasive positive pressure ventilation; PA: physical activity; HBPR: home-based pulmonary rehabilitation.

There were significantly greater changes in steps·day^−1^ compared to usual care for CBPR (Δ+680, 95% CI 12–1348 steps·day^−1^), CBPR+noninvasive positive pressure ventilation (NIPPV) (Δ+1787, 95% CI 107–3467 steps·day^−1^), CBPR+nutrition (Δ+1384, 95% CI 409–2359 steps·day^−1^), CBPR+physical activity promotion (Δ+1376, 95% CI 608–2144 steps·day^−1^) and HBPR (Δ+1252, 95% CI 332–2172 steps·day^−1^) (figure 2a).

CBPR+physical activity promotion was the only pulmonary rehabilitation-based intervention that resulted in a significantly greater change in steps per day when compared to CBPR alone (Δ+696, 95% CI 217–1176 steps·day^−1^), and that had a lower 95% confidence interval that surpassed the MID compared to usual care (95% CI 608–2144) (figure 2b).

### NMA: time spent in moderate-to-vigorous physical activity (min·day^−1^)

12 studies [27, 28, 56, 64, 66, 76, 78, 79, 81, 84, 87, 88] (n=1151) were included in the NMA for pre–post changes in average time spent in MVPA (min·day^−1^) in COPD. This consisted of six interventions and 15 pairwise comparisons (six of which were direct data comparisons; supplementary material E). No differences between direct and indirect information were observed (supplementary material B), and all comparisons between interventions are shown in supplementary material H.

**FIGURE 3:**
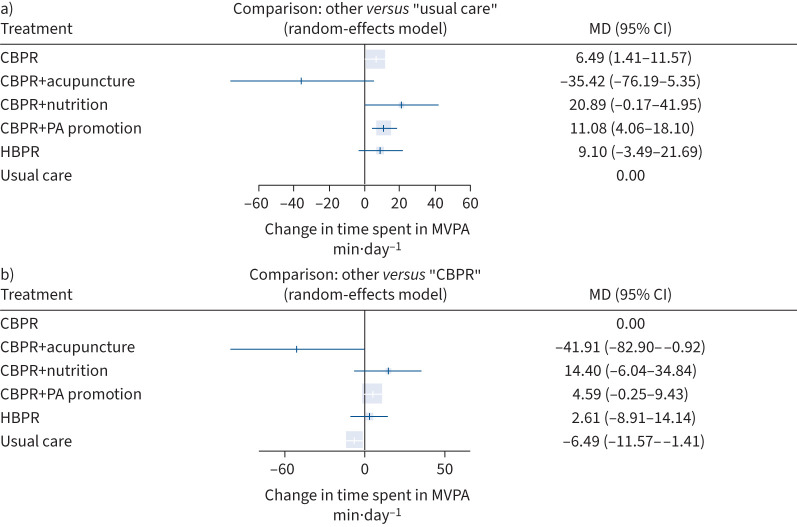
Forest plot comparing change in time spent in moderate to vigorous physical activity (MVPA; min·day^−1^) following pulmonary rehabilitation-based interventions for people with COPD with a) usual care and b) centre-based pulmonary rehabilitation (CBPR) (k=12, n=1151). MD: mean difference; PA: physical activity; HBPR: home-based pulmonary rehabilitation.

Significantly greater changes in time spent in MVPA were observed for CBPR (Δ+6.49, 95% CI 1.41–11.57 min·day^−1^) and CBPR+physical activity promotion (Δ+11.08, 95% CI 4.06–18.10 min·day^−1^) compared to usual care (figure 3a).

No significantly greater increases in time spent in MVPA were observed for any pulmonary rehabilitation-based intervention compared to CBPR alone (figure 3b).

### NMA: sedentary time (min·day^−1^)

Eight studies [27, 28, 76, 78, 79, 81, 84, 87] (n=881) were included in the NMA for pre–post changes in sedentary time (min·day^−1^) in COPD. This consisted of four interventions and six pairwise comparisons (three of which were direct data comparisons; supplementary material E). It was not possible to calculate differences between direct and indirect information within this NMA due to the low number of studies reporting this outcome (supplementary material B). All comparisons between interventions are shown in supplementary material H.

**FIGURE 4:**
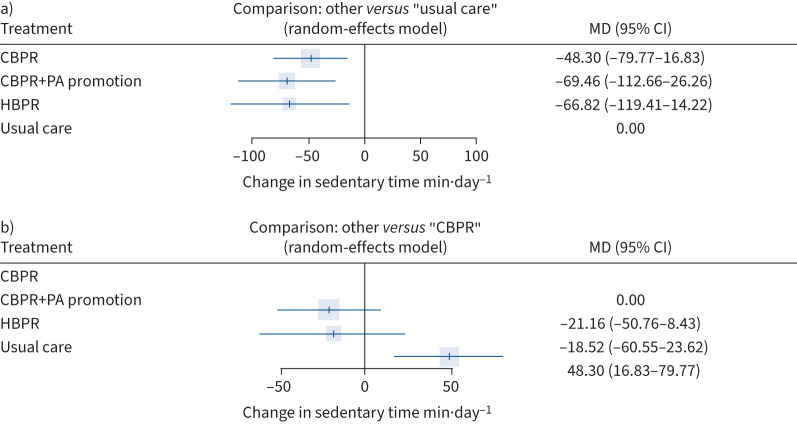
Forest plot comparing change in sedentary time (min·day^−1^) following pulmonary rehabilitation-based interventions for people with COPD with a) usual care and b) centre-based pulmonary rehabilitation (CBPR) (k=8, n=881). MD: mean difference; PA: physical activity; HBPR: home-based pulmonary rehabilitation.

Compared to usual care, significantly greater decreases in sedentary time were observed for CBPR (Δ−48.30, 95% CI −79.77– −16.83 min·day^−1^), CBPR+physical activity promotion (Δ−69.46, 95% CI −112.66– −26.26 min·day^−1^) and HBPR (Δ−66.82, 95% CI −119.41– −14.22 min·day^−1^; figure 4a).

There were no significant differences in changes in sedentary time following any pulmonary rehabilitation-based intervention compared to CBPR (figure 4b).

### Sensitivity analyses

Sensitivity analyses were conducted by using a correlation coefficient of 0.5 in place of the calculated correlation coefficient when imputing standard deviation (supplementary material I). This changed the standard deviation values for six studies [52, 57, 58, 79, 86] used within the daily step count NMA, and two changes from the results presented in figure 2 were observed, as follows. 1) CBPR+NIPPV failed to reach statistical significance compared to usual care, and 2) CBPR+nutrition resulted in significantly greater changes in steps per day compared to CBPR alone. The standard deviation values were changed in two studies [78, 79] reporting both MVPA and sedentary time, with the only observed change from initial analyses being the addition of CBPR+nutrition resulting in significantly greater changes in MVPA compared to usual care.

Findings were consistent between studies reporting daily step count and MVPA as primary and secondary outcomes (supplementary material J). We were unable to perform a sensitivity analysis for sedentary time, as no study assessed this as a primary outcome.

It was not possible to perform sensitivity analyses by removing studies classified as high risk of bias due to the limited number of remaining studies (see Risk-of-bias assessment).

### Risk-of-bias assessment

In accordance with the RoB2 tool, for the 44 studies using device-based measures of movement behaviours, one (2%) study was deemed to be low risk of bias and 29 (66%) studies were at high risk of bias (supplementary material K). For the nine studies using self-reported measures of movement behaviours, no studies were deemed to be low risk of bias and seven (78%) studies were at high risk of bias (supplementary material L). The most common domain resulting in studies being classified as at high risk of bias was domain 3 (“missing outcome data”) (k=28 (64%) out of 44).

### Quality-of-evidence assessment

The evidence from all thee outcomes of interest within this review (daily step count, time spent in MVPA, sedentary time) were deemed to be of “low certainty” (supplementary material M). Downgrading was due to high overall risk of bias, as well as imprecision due to most articles including these outcomes as secondary outcomes.

### Quality-of-reporting assessments

Of the 46 studies, all (100%) reported six or more out of 12 items from the TIDieR checklist, with 30 (65%) studies reporting nine or more out of 12 items and four (9%) studies reporting all 12 items (supplementary material N). All studies reported item 1 (“brief name”), item 2 (“why”) and item 8 (“when and how much”). Item 10 (“modifications”) was reported in the fewest studies (k=5; 11%).

Full details regarding the quality-of-reporting of device deployment for each included study are shown in the supplementary material O. Of the 44 articles using device-based measures of movement behaviours, the most frequent period of wear time was 7 days (k=33 (75.0%) out of 44). 17 (38.6%) studies did not report whether there was a valid wear time requirement, with the most frequent values in studies that did report this being 8 h or 10 h (both k=8 (18.2%) out of 44), followed by 12 h (k=5 (11.4%) out of 44). Studies predominantly reported valid wear time requirements as minimum values (21 (47.7%) out of 44) as opposed to fixed windows (k=4 (9.1%) out of 44), with the remaining 19 studies not reporting valid wear time requirements (k=19 (43.2%) out of 44). 21 (47.7%) studies did not report how many valid days were required to be included in the analysis. The most frequently reported number of valid days required were 4 days (11 (25%) out of 44). The valid days required were reported as minimum values in 19 (43.2%) studies, fixed in two (4.5%) studies and not specified in the remaining 23 (52.3%) studies.

Non-wear detection or a description of the identification of missing data was reported in three (6.8%) studies. Four (9.1%) studies reported average wear time values, with waking wear time calculable in nine (20.5%) studies, and not reported/calculable in the remaining 28 (79.5%) studies.

Four (40%) out of 10 possible studies did not report whether real-time feedback from devices were provided to the participants during the data collection period.

The most frequent questionnaire recall periods were 7 days (three (33.3%) out of nine) and 4 weeks (two (22.2%) out of nine; supplementary material P). Details regarding how missing questionnaire data were handled were only reported in one study [51], which stated that an intention-to-treat analysis method was followed. A further study [65] reported reasons for missing questionnaire data.

## Discussion

### Summary of main findings

In this first systematic review and NMA of changes in 24-h movement behaviours (physical activity, sedentary behaviour and sleep) in response to pulmonary rehabilitation-based interventions in CRD, CBPR alone was found to be superior to usual care (no pulmonary rehabilitation) for people with COPD in increasing volume-related physical activity (daily step count), increasing intensity-related physical activity (time spent in MVPA) and reducing sedentary behaviour (sedentary time). The addition of physical activity promotion to CBPR programmes was the only pulmonary rehabilitation-based intervention superior to CBPR alone at increasing volume-related physical activity, with no pulmonary rehabilitation-based intervention superior to CBPR at increasing MVPA or reducing sedentary behaviour. The effect of pulmonary rehabilitation-based interventions on sleep remains unclear due to the lack of randomised trials assessing this outcome. The high risk of bias within included studies and low certainty of evidence for these outcomes highlight the need for these findings to be viewed with caution.

### Interpretation of findings

The present systematic review and NMA provides insight into the specific pulmonary rehabilitation-based interventions that are superior in eliciting an increase in physical activity and/or a reduction in sedentary behaviour in COPD. CBPR alone was superior to usual care (no pulmonary rehabilitation) in increasing daily step count (mean change: +680 steps) in line with the MID of 600–1100 steps·day^−1^ [93], but with a lower 95% confidence interval of only 12 steps·day^−1^.

In keeping with the observed increase in volume-related physical activity following CBPR, the present study found reduced time spent sedentary. Given that most steps are taken at a low intensity and the strong relationship between light intensity physical activity and sedentary behaviour [94, 95], it is intuitive to expect sedentary time to be displaced by the additional time spent in physical activity.

In addition to CBPR resulting in a greater volume of physical activity, an increased time spent in MVPA was also observed. MVPA in included studies was examined from an absolute intensity perspective, whereby the threshold used to define MVPA intensity (*e.g.* ≥1952 counts·min^−1^ (ActiGraph)) [96] was applied to all individuals, regardless of their physical capacity. In CRD populations, typically characterised by poor exercise capacity, improvements in MVPA may be driven by the well-established gains in exercise capacity following CBPR [19]. At baseline, these absolute intensity thresholds for MVPA may not capture physical activity performed at a relatively high intensity [97, 98]. Therefore, it is unclear whether, and to what extent, improvements in MVPA are driven by behaviour change and/or greater exercise capacity following pulmonary rehabilitation. One way to address this might be to generate relative intensity thresholds by synchronising accelerometry with tests of walking exercise capacity [97, 99]. Within the present study, the relative perception of physical activity, either through device-based assessment or by self-report, could not be explored.

The addition of a physical activity promotion intervention to CBPR resulted in a superior increase in daily step count compared to CBPR alone (+696, 95% CI 217–1176 steps·day^−1^). The effectiveness of physical activity promotion interventions to increase daily steps is supported by previously published pairwise meta-analyses [29–31, 100–102], with the mean falling above the MID [93], but not the lower 95% confidence interval. However, when compared to usual care, this was the only intervention where the lower 95% confidence interval was above the MID [93]. Although the addition of physical activity promotion interventions to CBPR programmes resulted in greater time spent in MVPA compared to usual care, no evidence of superiority over CBPR alone was observed. Physical activity promotion adjuncts in the included studies mainly comprised pedometer-based interventions, thus targeting the volume of physical activity over the intensity. Not all steps are created equal, but if the goal is to get people moving more, then such adjuncts appear a viable option in the context of CBPR. Those with sufficient exercise capacity are likely to be a good target for behavioural interventions implemented alongside pulmonary rehabilitation, which aim to further enhance physical activity [22, 103].

Sleep was the least assessed movement behaviour within included articles [88–90]. This behaviour should not be overlooked in relation to pulmonary rehabilitation research and in the context of physical activity and sedentary behaviour. Poor sleep quality is associated with lower physical activity levels the following day in COPD patients [104]. With strategies to promote physical activity levels in CRD focusing on actions during the day, additional efforts in promoting sleep quality may facilitate further increases in physical activity [104].

It is important to evaluate interventions such as pulmonary rehabilitation from a 24-h perspective, which allows for a greater understanding on how these interventions influence behaviour. Only two articles [89, 90] within the present review assessed all three behaviours that make up a 24-h day (physical activity, sedentary behaviour and sleep), with only one article [89] reporting time spent in each behaviour. The authors of this article suggest that the limited evidence for improved physical activity following pulmonary rehabilitation interventions may relate to the analysis approach, rather than to a true absence of effect [89]. The compositional data analysis employed by Burge *et al*. [89] accounts for the fact that these components are bound by the 24 h that comprise each day. The results of this analysis showed a reduction in sedentary time relative to time in sleep, light intensity physical activity and MVPA, highlighting the interplay between movement behaviours.

### Limitations and considerations for future research

There are several considerations when interpreting the results of the present study. Our NMAs maximised the currently available data to compare (directly and indirectly) multiple pulmonary rehabilitation-based interventions against common comparators, usual care and CBPR, to identify the interventions superior in changing physical activity and sedentary behaviour. The findings from these NMAs only apply to COPD as there were insufficient data to allow us to perform NMAs for these outcomes in other CRDs. The present review identified large variation in the interventions and comparator groups tested, supporting the use of NMA over meta-analyses. In previous meta-analyses, it was not possible to unpack which types of interventions are more or less superior to a comparison group. NMA combines direct and indirect comparisons of three or more interventions simultaneously across a network of studies, yielding more precise estimates of the intervention effects compared with a single (direct or indirect) estimate [105, 106]. Our NMA found that CBPR was superior to usual care in increasing steps per day, but that the addition of physical activity promotion to CBPR was superior to both usual care and CBPR. Given the importance of having different pulmonary rehabilitation-based intervention to tailor patient care, we recommend future work to employ NMAs where possible.

The present review has also highlighted a wide range of measurement approaches and outcomes for physical activity and sedentary behaviour. This limited the current NMA to trials reporting device-based measures of steps per day, MVPA and sedentary time. Behind the data for these behaviours lay differences in measurement tools, data processing (*e.g.* valid day criteria), and quality of reporting between studies. These variations have also been identified by a previous review specifically focusing on objectively measured physical activity outcomes in COPD clinical trials [23]. Ideally, data would be pooled and harmonised to remove any variation in the data between studies [107]. Future trials of pulmonary rehabilitation measuring physical activity, sedentary behaviour and/or sleep, using devices such as accelerometers, should measure acceleration directly in the International System of Units (*e.g.* gravitational units) as this would allow direct comparisons between devices and studies [107]. The prospect of retrospective harmonisation of existing accelerometer data in trials of pulmonary rehabilitation remains quite limited, with the present review and previous work able to extract only device-specific outcomes [16, 108]. If the latest generation of accelerometers (*e.g.* ActiGraph, GENEActiv, Axivity) are adopted, it may then be possible to develop an international dataset of pooled accelerometer data in pulmonary rehabilitation trials. Open-source resources such as GGIR [109] are readily available to process and analyse raw acceleration data [107, 110].

Included studies were almost exclusively categorised as “at high risk of bias” or as “some concerns”, with missing outcome data (for physical activity, sedentary behaviour and/or sleep) the most common RoB2 domain classified as “at high risk of bias”. Furthermore, all three outcomes of interest were deemed to be of “low certainty of the evidence” predominantly due to the high risk of bias. The results presented in this review must therefore be interpreted with caution. The pulmonary rehabilitation-based interventions were generally well reported based on the TIDieR checklist. In most included studies, the quality of reporting for physical activity, sedentary behaviour and sleep outcome measures was not sufficient to allow future studies to replicate their approach. Items relating to the reporting of movement behaviour measurement used in the present review as well as from previous work [16, 41, 42] should be used to guide reporting in future trials.

Although the NMA approach allowed for the separation of pulmonary rehabilitation-based interventions and indirect comparisons, there was still significant variation within interventions, including duration and frequency of programmes, exercises used and level of supervision. Component NMA may offer a solution to this limitation, and has previously been utilised to determine the effect of exercise training programme designs on exercise capacity in COPD [34]. The use of this analysis approach is likely to lead to a deeper understanding of which pulmonary rehabilitation-based programme designs impact physical activity, sedentary behaviour, and sleep by considering the differing effects of programme designs within intervention types. The limited studies assessing these outcomes meant that we were unable to perform a component NMA within the present review.

The lack of trials assessing sleep prevented a NMA for this behaviour and highlights another important area for future research. Physical activity, sedentary behaviour and sleep were largely explored in isolation in included trials, preventing the synergistic change following pulmonary rehabilitation being explored, *e.g.* if more time is spent in physical activity, to what extent is time in sedentary behaviour or sleep displaced? A compositional approach to changes in 24-h movement behaviours following pulmonary rehabilitation has not been widely utilised [89]. High-quality studies with well-reported measurement and interventions, with 24-h movement behaviours as the primary outcome(s) are needed.

### Conclusion

This is the first NMA to assess the effect of pulmonary rehabilitation-based interventions of 24-h movement behaviours (physical activity, sedentary behaviour and sleep). Our findings suggest that the addition of a physical activity promotion intervention to pulmonary rehabilitation may significantly increase the volume of physical activity (*i.e.* daily step count); however, CBPR alone may be sufficient at both increasing the intensity of physical activity (*i.e.* time spent in MVPA) and reducing sedentary time. However, included studies were mostly at high risk of bias and lower limits of confidence intervals were below the MID, therefore these results need to be viewed with caution. Variations in pulmonary rehabilitation-based interventions, the wide range of measurement tools and outcome variables, poor reporting quality of measurement approaches, and lack of combined assessment of physical activity, sedentary behaviour and sleep should be addressed in future trials of pulmonary rehabilitation examining 24-h movement behaviours.

**Points for clinical practice and questions for future research**

- Different pulmonary rehabilitation interventions lead to different responses in physical activity and sedentary behaviour.
- Centre-based pulmonary rehabilitation can increase both volume-related (daily step count) and intensity-related physical activity (time spent in moderate-to-vigorous physical activity).
- The addition of physical activity promotion can further increase daily step count compared to centre-based pulmonary rehabilitation alone.
- There is a need for more studies investigating the effect of pulmonary rehabilitation-based interventions on sleep quality to determine the interplay between physical activity, sedentary behaviour and sleep in response to pulmonary rehabilitation.

## Supplementary material

10.1183/16000617.0225-2023.Supp1 **Please note:** supplementary material is not edited by the Editorial Office, and is uploaded as it has been supplied by the author.
Supplementary material ERR-0225-2023.SUPPLEMENT
