# Supplementary material for: Changes in physical activity, sedentary behaviour and sleep following pulmonary rehabilitation: a systematic review and network meta-analysis
Source: Eur Respir Rev. 2024 Apr 10;33(172):230225. doi: 10.1183/16000617.0225-2023 (PMC11004771; doi:10.1183/16000617.0225-2023)
Supplement: Supplementary file 1 [file ERR-0225-2023.SUPPLEMENT.pdf]

Supplementary material

Contents

Supplementary material A – Search strategies ... 2

Supplementary material B – Inconsistency results for network meta-analyses for a) daily step count, b) time spent in moderate-to-vigorous physical activity, and c) sedentary time ... 8

Supplementary material C – Full extraction of results pertaining to 24-hour movement behaviours ... 14

Supplementary material D – Details of intervention and comparator groups for individual studies ... 31

Supplementary material E – Network plot for a) all included articles (n=48), and those reporting changes in b) daily step count, c) time spent in moderate-to-vigorous physical activity, and d) sedentary time ... 60

Supplementary material F – Radial plot showing the number of descriptive outcomes reported for physical activity (PA), sedentary behaviour (SB) and sleep quality, connected to the measurement tools used to generate them ... 62

Supplementary material G – Primary vs secondary outcomes ... 63

Supplementary material H – All comparisons between interventions for changes in a) daily step count, b) time spent in MVPA, and c) sedentary time. ... 65

Supplementary material I – Sensitivity analyses: correlation coefficient of 0.5 when imputing SDs for a) daily step count, b) time spent in moderate-to-vigorous physical activity, and c) sedentary time ... 68

Supplementary material J – Sensitivity analyses: Primary vs secondary outcomes for a) daily step count, and b) time spent in moderate-to-vigorous physical activity ... 71

Supplementary material K - Risk of Bias 2 results for studies using device-based measures of movement behaviours ... 72

Supplementary material L - Risk of Bias 2 results for studies using questionnaire-based measures of movement behaviours ... 73

Supplementary material M - GRADE results for a) daily step count, b) time spent in MVPA, and c) sedentary time ... 74

Supplementary material N – TIDieR results for individual studies (1, provided; 0, not provided) ... 77

Supplementary material O – Quality and reporting details of device deployment for studies using device-based measures of movement behaviours ... 79

Supplementary material P – Quality and reporting details of questionnaires for studies using questionnaire-based measures of movement behaviours ... 92

Supplementary material A – Search strategies

MEDLINE

(

(MH "pulmonary disease, chronic obstructive") OR (MH "Bronchitis, Chronic") OR (MH "Tuberculosis, Pulmonary") OR (MH "Respiratory Tract Diseases") OR (MH "Hypertension, Pulmonary") OR (MH "Idiopathic Interstitial Pneumonias") OR (MH "Lung Diseases, Interstitial") OR (MH "Pulmonary Fibrosis") OR (MH "Alveolitis, Extrinsic Allergic")

OR

TI("Chronic obstructive pulmonary disease" OR "COPD" OR "Emphysema" OR "Lung disease*" OR "asthma" OR "Idiopathic pulmonary fibrosis" OR "Interstitial lung disease" OR "Idiopathic fibrosing alveolitis")

OR

AB("Chronic obstructive pulmonary disease" OR "COPD" OR "Emphysema" OR "Lung disease*" OR "asthma" OR "Idiopathic pulmonary fibrosis" OR "Interstitial lung disease" OR "Idiopathic fibrosing alveolitis")

)

AND

(

(MH rehabilitation) OR (MH rehabilitation nursing)

OR

TI("rehabilitation cent*" OR rehab* OR telerehabilitation OR "prescribed exercise" OR "prescribed activity" OR "Pulmonary Rehabilitation" OR "Lung Rehabilitation" OR "Respiratory Rehabilitation")

OR

AB("rehabilitation cent*" OR rehab* OR telerehabilitation OR "prescribed exercise" OR "prescribed activity" OR "Pulmonary Rehabilitation" OR "Lung Rehabilitation" OR "Respiratory Rehabilitation")

)

AND

(

(MH Motor Activity) OR (MH Leisure Activities) OR (MH Physical Fitness) OR (MH Sports) OR (MH Recreation) OR (MH Exercise) OR (MH PhysicalFitness) OR (MH Walking) OR (MH Sedentary Behavior) OR (MH Screen Time) OR (MH Television) OR (MH videogames) OR (MH Social Media) OR (MH internet) OR (MH Smartphone) OR (MH Computers, Handheld) OR (MH Cell Phone) OR (MH computers) OR (MH reading) OR (MH writing) OR (MH Automobile Driving) OR (MH bicycling) OR (MH Physical Exertion) OR (MH Energy Metabolism) OR (MH Sleep)

OR

TI("aerobic activit*" OR "motor activit*" OR "leisure activit*" OR "habitual activit*" OR "physical activit*" OR "physical inactivit*" OR "physical fitness" OR sport* OR recreation OR exercis* OR

fitness OR walk* OR “step count” OR sedentar* OR sitting OR reclining OR "lying down" OR leisure time behavio* OR “small screen” OR television OR TV OR “electronic media” OR "social media" OR “reading time” OR “writing time” OR “studying time” OR “driving time” OR “passive transportation” OR “active transport” OR “active travel” OR bicycl* OR cycling OR energy expenditure OR sleep*)

OR

AB(“aerobic activit*” OR “motor activit*” OR “leisure activit*” OR “habitual activity” OR “physical activity” OR “physical inactivity” OR “physical fitness” OR sport* OR recreation OR exercis* OR fitness OR walk* OR “step count” OR sedentar* OR sitting OR reclining OR "lying down" OR leisure time behavio* OR “small screen” OR (television or TV) OR electronic media OR "social media" OR “reading time” OR “writing time” OR “studying time” OR “driving time” OR “passive transportation” OR “active transport” OR “active travel” OR bicycl* OR cycling OR energy expenditure OR sleep* OR “sleep hour*” OR “sleep duration” OR “sleep time” OR “sleep length” OR “sleep period” OR “sleep span”)

)

NOT

(

TI(protocol OR “systematic review” OR “scoping review” OR “meta-analysis” OR qualitative OR cross-sectional OR “case study” OR cohort OR “case report”)

OR

AB(protocol OR “systematic review” OR “scoping review” OR “meta-analysis” OR qualitative OR cross-sectional OR “case study” OR cohort OR “case report”)

)

CINAHL Complete

(

(MH "pulmonary disease, chronic obstructive") OR (MH "Bronchitis, Chronic") OR (MH "Tuberculosis, Pulmonary") OR (MH "Respiratory Tract Diseases") OR (MH "Hypertension, Pulmonary") OR (MH "Idiopathic Interstitial Pneumonias") OR (MH "Lung Diseases, Interstitial") OR (MH "Pulmonary Fibrosis") OR (MH "Alveolitis, Extrinsic Allergic")

OR

TI(“Chronic obstructive pulmonary disease” OR “COPD” OR “Emphysema” OR “Lung disease*” OR “asthma” OR “Idiopathic pulmonary fibrosis” OR “Interstitial lung disease” OR “Idiopathic fibrosing alveolitis”)

OR

AB(“Chronic obstructive pulmonary disease” OR “COPD” OR “Emphysema” OR “Lung disease*” OR “asthma” OR “Idiopathic pulmonary fibrosis” OR “Interstitial lung disease” OR “Idiopathic fibrosing alveolitis”)

)

AND

(

(MH “rehabilitation”)

OR

TI(“rehabilitation cent*” OR rehab* OR telerehabilitation OR “prescribed exercise” OR “prescribed activity” OR “Pulmonary Rehabilitation” OR “Lung Rehabilitation” OR “Respiratory Rehabilitation”)

OR

AB(“rehabilitation cent*” OR rehab* OR telerehabilitation OR “prescribed exercise” OR “prescribed activity” OR “Pulmonary Rehabilitation” OR “Lung Rehabilitation” OR “Respiratory Rehabilitation”)

)

AND

(

(MH “Motor Activity”) OR (MH “Leisure Activities”) OR (MH “Physical Fitness”) OR (MH “Sports”) OR (MH “Recreation”) OR (MH “Exercise”) OR (MH “Physical Fitness”) OR (MH “Walking”) OR (MH “Lifestyle, Sedentary”) OR (MH “Screen Time”) OR (MH “Television”) OR (MH “Video Games”) OR (MH “Social Media”) OR (MH “internet”) OR (MH “Smartphone”) OR (MH “Cellular Phone”) OR (MH “Computers, Portable”) OR (MH “Computers, Hand-Held”) OR (MH “reading”) OR (MH “writing”) OR (MH “Automobile Driving”) OR (MH “Cycling”) OR (MH “Exertion”) OR (MH “Energy Metabolism”) OR (MH “Sleep”)

OR

TI(“aerobic activit*” OR “motor activit*” OR “leisure activit*” OR “habitual activit*” OR “physical activit*” OR “physical inactivit*” OR “physical fitness” OR sport* OR recreation OR exercis* OR fitness OR walk* OR “step count” OR sedentar* OR sitting OR reclining OR "lying down" OR “leisure time behavio*” OR “small screen” OR television OR tv OR “electronic media” OR "social

media" OR Computer OR “reading time” OR “writing time” OR “studying time” OR “driving time” OR “passive transportation” OR “active transport” OR “active travel” OR bicycl* OR cycling OR energy expenditure OR sleep*)

OR

AB(“aerobic activit*” OR “motor activit*” OR “leisure activit*” OR “habitual activit*” OR “physical activit*” OR “physical inactivit*” OR “physical fitness” OR sport* OR recreation OR exercis* OR fitness OR walk* OR “step count” OR sedentar* OR sitting OR reclining OR "lying down" OR “leisure time behavio*” OR “small screen” OR television OR tv OR “electronic media” OR "social media" OR Computer OR “reading time” OR “writing time” OR “studying time” OR “driving time” OR “passive transportation” OR “active transport” OR “active travel” OR bicycl* OR cycling OR energy expenditure OR sleep*)

)

NOT

(

TI(protocol OR “systematic review” OR “scoping review” OR “meta-analysis” OR qualitative OR cross-sectional OR “case study” OR cohort OR “case report”)

OR

AB(protocol OR “systematic review” OR “scoping review” OR “meta-analysis” OR qualitative OR cross-sectional OR “case study” OR cohort OR “case report”)

)

APA PsycInfo

(

TI(“Chronic obstructive pulmonary disease” OR “COPD” OR “Emphysema” OR "Chronic bronchitis" OR "Pulmonary Tuberculosis" OR "Respiratory Tract Diseases" OR "Pulmonary Hypertension " OR Idiopathic Interstitial Pneumonias" OR " Interstitial Lung Diseases" OR "Pulmonary Fibrosis" OR " Extrinsic Allergic Alveolitis" OR “Lung disease*” OR “asthma” OR “Idiopathic pulmonary fibrosis” OR “Interstitial lung disease” OR “Idiopathic fibrosing alveolitis”)

OR

AB(“Chronic obstructive pulmonary disease” OR “COPD” OR “Emphysema” OR "Chronic bronchitis" OR "Pulmonary Tuberculosis" OR "Respiratory Tract Diseases" OR "Pulmonary Hypertension " OR Idiopathic Interstitial Pneumonias" OR " Interstitial Lung Diseases" OR "Pulmonary Fibrosis" OR " Extrinsic Allergic Alveolitis" OR “Lung disease*” OR “asthma” OR “Idiopathic pulmonary fibrosis” OR “Interstitial lung disease” OR “Idiopathic fibrosing alveolitis”

)

AND

(

TI(“rehabilitation cent*” OR rehab* OR telerehabilitation OR “prescribed exercise” OR “prescribed activity” OR “Pulmonary Rehabilitation” OR “Lung Rehabilitation” OR “Respiratory Rehabilitation”)

OR

AB(“rehabilitation cent*” OR rehab* OR telerehabilitation OR “prescribed exercise” OR “prescribed activity” OR “Pulmonary Rehabilitation” OR “Lung Rehabilitation” OR “Respiratory Rehabilitation”)

)

AND

(

TI(“motor activity” OR “leisure activit*” OR “aerobic activit*” OR “motor activit*” OR “leisure activit*” OR “habitual activity” OR “physical activity” OR “physical inactivity” OR “physical fitness” OR “physical fitness” OR sport* OR recreation OR exercis* OR fitness OR walk* OR “step count” OR sedentar* OR sitting OR reclining OR "lying down" OR “leisure time behavio*” OR “screen time” OR “small screen” OR television OR TV OR “electronic media” OR videogames OR Internet OR "social media" OR smartphone OR “cell phone” OR computer OR writing OR “reading time” OR “writing time” OR “studying time” OR “driving time” OR “automobile driving” OR “passive transportation” OR “active transport” OR “active travel” OR bicycl* OR cycl* OR “physical exertion” OR “energy expenditure” OR “energy metabolism” OR sleep*)

OR

AB(“motor activity” OR “leisure activit*” OR “aerobic activit*” OR “motor activit*” OR “leisure activit*” OR “habitual activity” OR “physical activity” OR “physical inactivity” OR “physical fitness” OR “physical fitness” OR sport* OR recreation OR exercis* OR fitness OR walk* OR “step count” OR sedentar* OR sitting OR reclining OR "lying down" OR “leisure time behavio*” OR “screen time” OR “small screen” OR television OR TV OR “electronic media” OR videogames OR Internet OR "social media" OR smartphone OR “cell phone” OR computer OR writing OR “reading time” OR “writing time” OR “studying time” OR “driving time” OR “automobile driving” OR

“passive transportation” OR “active transport” OR “active travel” OR bicycl* OR cycl* OR “physical exertion” OR “energy expenditure” OR “energy metabolism” OR sleep*)

)

NOT

(

TI(protocol OR “systematic review” OR “scoping review” OR “meta-analysis” OR qualitative OR cross-sectional OR “case study” OR cohort OR “case report”)

OR

AB(protocol OR “systematic review” OR “scoping review” OR “meta-analysis” OR qualitative OR cross-sectional OR “case study” OR cohort OR “case report”)

)

Supplementary material B – Inconsistency results for network meta-analyses for a) daily step count, b) time spent in moderate-to-vigorous physical activity, and c) sedentary time

a) Daily step count

	Comparison	No.Studies	NMA	Direct	Indirect	Difference	Diff_95CI_ lower	Diff_95CI_ upper	pValue
1	CBPR:CBPR water-based	1	-676.00	-676.00	NA	NA	NA	NA	NA
2	CBPR:CBPR+acupuncture	1	794.17	451.00	2166.68	-1715.68	-4641.70	1210.33	0.25
3	CBPR:CBPR+downhill walking	1	-176.00	-176.00	NA	NA	NA	NA	NA
4	CBPR:CBPR+enhanced education	1	414.00	414.00	NA	NA	NA	NA	NA
5	CBPR:CBPR+medication	1	943.00	943.00	NA	NA	NA	NA	NA
6	CBPR:CBPR+NIPPV	1	-1107.00	-1107.00	NA	NA	NA	NA	NA
7	CBPR:CBPR+nutrition	3	-704.45	-704.45	NA	NA	NA	NA	NA
8	CBPR:CBPR+PA promotion	8	-696.30	-728.66	-74.50	-654.16	-2865.31	1556.98	0.56
9	CBPR:HBPR	5	-572.26	-625.67	323.46	-949.13	-3869.23	1970.97	0.52
10	CBPR:Usual care	3	679.87	756.42	234.43	522.00	-1366.50	2410.49	0.59
11	CBPR water-based:CBPR+acupuncture	0	1470.17	NA	1470.17	NA	NA	NA	NA
12	CBPR water-based: CBPR+downhill walking	0	500.00	NA	500.00	NA	NA	NA	NA
13	CBPR water-based: CBPR+enhanced education	0	1090.00	NA	1090.00	NA	NA	NA	NA

14	CBPR water-based: CBPR+medication	0	1619.00	NA	1619.00	NA	NA	NA	NA
15	CBPR water-based: CBPR+NIPPV	0	-431.00	NA	-431.00	NA	NA	NA	NA
16	CBPR water-based: CBPR+nutrition	0	-28.45	NA	-28.45	NA	NA	NA	NA
17	CBPR water-based:CBPR+PA promotion	0	-20.30	NA	-20.30	NA	NA	NA	NA
18	CBPR water-based:HBPR	0	103.74	NA	103.74	NA	NA	NA	NA
19	CBPR water-based:Usual care	0	1355.87	NA	1355.87	NA	NA	NA	NA
20	CBPR+acupuncture:CBPR+ downhill walking	0	-970.17	NA	-970.17	NA	NA	NA	NA
21	CBPR+acupuncture:CBPR+ enhanced education	0	-380.17	NA	-380.17	NA	NA	NA	NA
22	CBPR+acupuncture: CBPR+medication	0	148.83	NA	148.83	NA	NA	NA	NA
23	CBPR+acupuncture: CBPR+NIPPV	0	-1901.17	NA	-1901.17	NA	NA	NA	NA
24	CBPR+acupuncture: CBPR+nutrition	0	-1498.62	NA	-1498.62	NA	NA	NA	NA
25	CBPR+acupuncture: CBPR+PA promotion	0	-1490.46	NA	-1490.46	NA	NA	NA	NA
26	CBPR+acupuncture:HBPR	0	-1366.42	NA	-1366.42	NA	NA	NA	NA
27	CBPR+acupuncture:Usual care	1	-114.30	-365.00	1669.11	-2034.11	-5503.20	1434.98	0.25
28	CBPR+downhill walking: CBPR+enhanced education	0	590.00	NA	590.00	NA	NA	NA	NA

29	CBPR+downhill walking: CBPR+medication	0	1119.00	NA	1119.00	NA	NA	NA	NA
30	CBPR+downhill walking: CBPR+NIPPV	0	-931.00	NA	-931.00	NA	NA	NA	NA
31	CBPR+downhill walking:CBPR+nutrition	0	-528.45	NA	-528.45	NA	NA	NA	NA
32	CBPR+downhill walking: CBPR+PA promotion	0	-520.30	NA	-520.30	NA	NA	NA	NA
33	CBPR+downhill walking:HBPR	0	-396.26	NA	-396.26	NA	NA	NA	NA
34	CBPR+downhill walking:Usual care	0	855.87	NA	855.87	NA	NA	NA	NA
35	CBPR+enhanced education:CBPR+medication	0	529.00	NA	529.00	NA	NA	NA	NA
36	CBPR+enhanced education:CBPR+NIPPV	0	-1521.00	NA	-1521.00	NA	NA	NA	NA
37	CBPR+enhanced education:CBPR+nutrition	0	-1118.45	NA	-1118.45	NA	NA	NA	NA
38	CBPR+enhanced education: CBPR+PA promotion	0	-1110.30	NA	-1110.30	NA	NA	NA	NA
39	CBPR+enhanced education: HBPR	0	-986.26	NA	-986.26	NA	NA	NA	NA
40	CBPR+enhanced education:Usual care	0	265.87	NA	265.87	NA	NA	NA	NA
41	CBPR+medication: CBPR+NIPPV	0	-2050.00	NA	-2050.00	NA	NA	NA	NA

42	CBPR+medication: CBPR+nutrition	0	-1647.45	NA	-1647.45	NA	NA	NA	NA
43	CBPR+medication: CBPR+PA promotion	0	-1639.30	NA	-1639.30	NA	NA	NA	NA
44	CBPR+medication:HBPR	0	-1515.26	NA	-1515.26	NA	NA	NA	NA
45	CBPR+medication:Usual care	0	-263.13	NA	-263.13	NA	NA	NA	NA
46	CBPR+NIPPV: CBPR+nutrition	0	402.55	NA	402.55	NA	NA	NA	NA
47	CBPR+NIPPV: CBPR+PA promotion	0	410.70	NA	410.70	NA	NA	NA	NA
48	CBPR+NIPPV:HBPR	0	534.74	NA	534.74	NA	NA	NA	NA
49	CBPR+NIPPV:Usual care	0	1786.87	NA	1786.87	NA	NA	NA	NA
50	CBPR+nutrition:CBPR+PA promotion	0	8.15	NA	8.15	NA	NA	NA	NA
51	CBPR+nutrition:HBPR	0	132.20	NA	132.20	NA	NA	NA	NA
52	CBPR+nutrition:Usual care	0	1384.32	NA	1384.32	NA	NA	NA	NA
53	CBPR+PA promotion:HBPR	0	124.04	NA	124.04	NA	NA	NA	NA
54	CBPR+PA promotion: Usual care	1	1376.17	1115.00	1502.22	-387.22	-2025.71	1251.26	0.64
55	HBPR:Usual care	1	1252.13	409.00	1358.13	-949.13	-3869.23	1970.97	0.52

b) Time spent in moderate-to-vigorous physical activity

	Comparison	No.Studies	NMA	Direct	Indirect	Difference	Diff_95CI_ lower	Diff_95CI_ upper	pValue
1	CBPR:CBPR+acupuncture	1	41.91	31.30	132.25	-100.95	-234.61	32.72	0.14
2	CBPR:CBPR+nutrition	1	-14.40	-14.40	NA	NA	NA	NA	NA
3	CBPR:CBPR+PA promotion	4	-4.59	-4.59	NA	NA	NA	NA	NA
4	CBPR:HBPR	4	-2.61	-2.61	NA	NA	NA	NA	NA
5	CBPR:Usual care	3	6.49	6.49	NA	NA	NA	NA	NA
6	CBPR+acupuncture: CBPR+nutrition	0	-56.31	NA	-56.31	NA	NA	NA	NA
7	CBPR+acupuncture: CBPR+PA promotion	0	-46.50	NA	-46.50	NA	NA	NA	NA
8	CBPR+acupuncture:HBPR	0	-44.52	NA	-44.52	NA	NA	NA	NA
9	CBPR+acupuncture:Usual care	1	-35.42	-37.30	468.28	-505.58	-1175.02	163.86	0.14
10	CBPR+nutrition: CBPR+PA promotion	0	9.81	NA	9.81	NA	NA	NA	NA
11	CBPR+nutrition:HBPR	0	11.79	NA	11.79	NA	NA	NA	NA
12	CBPR+nutrition:Usual care	0	20.89	NA	20.89	NA	NA	NA	NA
13	CBPR+PA promotion:HBPR	0	1.98	NA	1.98	NA	NA	NA	NA
14	CBPR+PA promotion:Usual care	0	11.08	NA	11.08	NA	NA	NA	NA
15	HBPR:Usual care	0	9.10	NA	9.10	NA	NA	NA	NA

c) Sedentary time

	Comparison	No.Studies	NMA	Direct	Indirect	Difference	Diff_95CI_ lower	Diff_95CI_ upper	pValue
1	CBPR:CBPR+PA promotion	3	21.16	21.16	NA	NA	NA	NA	NA
2	CBPR:HBPR	3	18.52	18.52	NA	NA	NA	NA	NA
3	CBPR:Usual care	2	-48.30	-48.30	NA	NA	NA	NA	NA
4	CBPR+PA promotion:HBPR	0	-2.64	NA	-2.64	NA	NA	NA	NA
5	CBPR+PA promotion:Usual care	0	-69.46	NA	-69.46	NA	NA	NA	NA
6	HBPR:Usual care	0	-66.82	NA	-66.82	NA	NA	NA	NA

Supplementary material C – Full extraction of results pertaining to 24-hour movement behaviours

Author (Year)	Experimental group			Control group		
	Baseline values	Post-intervention values	Change	Baseline values	Post-intervention values	Change
**Physical activity**						
**Step count (steps/day)**						
**ActiGraph**						
Armstrong (2021)	3450±2168	4426±2577	976±925<br><br>(data provided by author)	3446±2342	3406±2095	−40±626<br><br>(data provided by author)
Camillo (2020)	4567±2927	5027±3063	460±1899<br><br>(correlation coefficient input)	5032±2754	5316±2877	284±1785<br><br>(correlation coefficient input)
Cerdan-de-las-Heras (2021a)	13,629±5314	13,574±8973		11,883±5237	14,017±9663	
Cerdan-de-las-Heras (2021b)	8835±4387	8999±4241	165±4170<br><br>(data provided by author)	11,322±8465	11,985±10,928	663±4218<br><br>(data provided by author)
Cerdan-de-las-Heras (2022)	17,935±11,072	16,780±5100		14,282±7515	14,245±6551	
Cruz (2016)	7161.5±1708.1	10,440.0±4012.9	3279±2838<br><br>(correlation coefficient input)	6617.1±2914.2	6430.0±2613.1	−187±1771<br><br>(correlation coefficient input)
Geidl (2021)	5722.4±2948.6	6875.0±3229.5	1153±1972<br><br>(correlation coefficient input)	5934.5±3101.0	6679.5±3337.4	745±2048<br><br>(correlation coefficient input)
José (2021)	7340±4754		735.76±3114.20	6045±2731		−592.70±2032.41

Louvaris (2016)	4043±2484	5136±2866		3871±2526	3453±2493	
O'Neill (2018)	3305.6±1960.2	4768.2±2992.1	972±3230	3834.6±2245.5	3476.6±2307.9	4.3±663
Park (2020)	5223.68±2899.61	6546.77±2354.43		6756.26±2978.77	6890.39±2967.73	
Polkey (2018)	7992±3894	Mid-intervention: 8707±3549<br><br>2 months after intervention: 7907±3568		7005±3619	Mid-intervention: 7638±3379<br><br>2 months after intervention: 6558±3137	
van de Bool (2017)	4790.1±352.2	4866.4±479.0	76±385	4464.7±415.9	3841.9±393.4	−823±284
**SenseWear**						
Cedeño de Jesús (2022)	4578±3424	6591±3482		4793±3236	4824±3113	
Chaplin (2022)	5465±3013	6112±2464	647±2236<br><br>(data provided by author)	5300±3403	5409±3378	109±2001<br><br>(data provided by author)
Curtis (2016)	6685±4234		−382±2082	4883±2668		561±2528
Deering (2011)	PR+A = 3872±3719<br><br>PR = 2927±1989		PR+A = −194.6±1156<br><br>PR = 256.1±1391	2486±2588		169.9±630.7
Holland (2017)<br><br>*Change scores: means (95% CI)*	3836±2657		520 (−208 to 1249)<br><br>±1767 (SD calculated from 95% CI)	3670±2532		−160 (−798 to 478)<br><br>±1799 (SD calculated from 95% CI)
Horton (2021)<br><br>*Change scores: means (95% CI)*	3902±2120<br><br>(data from authors)	4976±3130<br><br>(data from authors)	1074 (289 to 1708)<br><br>±1756 (SD calculated from 95% CI)	3683±1820<br><br>(data from authors)	3219±1829<br><br>(data from authors)	−464 (−1445 to 517)<br><br>±2377 (SD calculated from 95% CI)
Jarosch (2020)<br><br>(reported as responders and non-responders in	Responders: 4854<br><br>Non-responders: 4926	Responders: 5104<br><br>Non-responders: 2923		Not reported	Not reported	

six-minute walk distance following PR)						
Lahham (2020)	5861±3051		303 (−1607 to 2215)<br><br>±5021 (SD calculated from 95% CI)	5181±3286		−106 (−1962 to 1749)<br><br>±4879 (SD calculated from 95% CI)
Nolan (2017)<br><br>*(median [first quartile, third quartile])*	3293 (1717, 5502)		272 (−342, 782)<br><br>386±2100 (mean±SD provided by author)	3456 (1567, 5925)		155 (−438, 867)<br><br>−6±1779 (mean±SD provided by author)
Pavitt (2020)<br><br>*(median [IQR])*	3507 (1605, 5314)		348 (−94, 1629)<br><br>637±2750 (mean±SD provided by author)	3791 (2271, 6885)		−329 (−915, 640)<br><br>−577±1943 (mean±SD provided by author)
Perez-Bogerd (2018)	5671±2598	3 months: 5721±2598<br><br>6 months: 5540±2598		7013±2598	3 months: 6593±2598<br><br>6 months: 6118±2598	
Rausch Osthoff (2021)	4987± 2751	5026± 2859	39±1777<br><br>(correlation coefficient input)	5581±3413	5651±3582	70±2218<br><br>(correlation coefficient input)
Wallaert (2020)<br><br>*Median (IQR)*	6263 (3937-10336)	6869 (5154-9162)		5396 (3522-7538)	Not Reported	
**Minimod (Dynaport)**						
Burtin (2015)<br><br>*Baseline: median (IQR)*	3408 (1732-5709)		576±2649<br><br>(data provided by author)	2574 (1592-4631)		394±845<br><br>(data provided by author)

**Dynaport MoveMonitor**						
Troosters (2018)<br><br>*(mean±SE)*	Self-management behaviour modification (SMBM) + placebo: 5507±188		SMBM + placebo: 922±325	Not reported		SMBM + tiotropium: 676±325<br>SMBM + tiotropium/ olodaterol: 1147±320<br><br>(above groups combined within RevMan for control group within network meta-analysis)<br><br>SMBM + tiotropium/ olodaterol + exercise: 594±314
**ActivePAL**						
Hansen (2020)<br><br>*Change scores: mean diff (95% CI)*	2779±1966		−116 (−503 to 270)<br><br>±1587 (SD calculated from 95% CI)	3422±2335		−400 (−803 to −2.3)<br><br>±1628 (SD calculated from 95% CI)
**Fitbit Flex/Flex2**						
Selzler (2021)	4272±339	4830±441	262±1761<br><br>(data provided by author)	4174±350	4943±454	676±2730<br><br>(data provided by author)
**Yamax pedometer**						
Aldhahir (2021)	4297 (1726, 7211)	5973 (2000, 6812)	197±813<br><br>(calculated from median [IQR] values)	2663 (1947, 4912)	2903 (1800, 4753)	49±1247<br><br>(calculated from median [IQR] values)
Altenburg (2015)<br><br>*(median [IQR])*	Total: 4292 (2182-6596)	Total: 5751 (3598-7238)	PR: −170±1942<br><br>PR+PA: 685±900	Total: 4132 (2979-6030)	Total: 4257 (3139-5696)<br><br>Primary: 4757 (3304-5858)	Usual care: −430±2473

	Primary care: 5961 (3788-8243)<br><br>Secondary care: 4820 (2526-6563)<br><br>PR+PA: 2276 (1883-4880)	Primary care: 6540 (5252-8619)<br><br>Secondary care: 5751 (3801-9701)<br><br>PR+PA: 4138 (2599-6413)	(calculated from median [IQR])	Primary: 4785 (2850-6757)<br><br>Secondary: 4285 (3609-6292)<br><br>PR: 3668 (2581-5389)	Secondary: 4221 (3318-6046)<br><br>PR: 3852 (2396-4964)	(calculated from median [IQR] for separate primary and secondary care groups then combined within RevMan)
de Blok (2006)<br><br>*(mean [95% CI])*	7 days: 2140 (1201-3079)<br><br>4 days: 2059 (1118-3000)<br><br>6 days: 2082 (1139-3025)	7 days: 3927 (2114-5741)<br><br>4 days: 3594 (1937-5251)<br><br>6 days: 3512 (1797-5227)	6 days: 1430±1333<br><br>(pre-post SDs calculated from 95% CI then correlation coefficient input for SD of change)	7 days: 2334 (1446-3242)<br><br>4 days: 2312 (1396-3227)<br><br>6 days: 2377 (1370-3384)	3554 (2155-4954)<br><br>2985 (1255-4716)<br><br>2832 (1530-4133)	6 days: 455±935<br><br>(pre-post SDs calculated from 95% CI then correlation coefficient input for SD of change)
Duiverman (2008)<br><br>*(median [IQR])*	1893 (591-3773)	2799 (891-6135)	1189±2604<br><br>(pre-post SDs calculated from IQR then correlation coefficient input for SD of change)	1680 (699-3538)	2093 (914-3155)	82±1322<br><br>(pre-post SDs calculated from IQR then correlation coefficient input for SD of change)
Effing (2011)	4472±2715		7 months: 478±2677<br><br>(calculated from mean±SE)	5224±3464		−87±2261<br><br>(calculated from mean±SE)
Felcar (2018)	6101±3591	3 months: 7100±3405<br><br>6 months: 7770±3629	6 months: 1669±2703<br><br>(calculated from 95% CI)	5891±3054	3 months: 6457±2949<br><br>6 months: 6884±3116	6 months: 993±1192<br><br>(calculated from 95% CI)
Nolan (2017)<br><br>*(median [first quartile, third quartile])*	2329 (1416, 4449)		727 (−1493, 3119)	2531 (1440, 4062)		829 (−1187, 2534)

Polgar (2021)<br><br>*Combined study arms of Nolan (2017)*<br><br>*(median [IQR])*	2418 (1440-42610		420 (−258-1582)			
**OMRON Walking Style X Pocket**						
Varas (2018)	7153±3607		3158±2191	5888±2121		−16±2795
**Time in moderate-to-vigorous PA (MVPA; min/day [e.g. time in >3METs, time in ≥3METs])**						
**ActiGraph**						
Armstrong (2021)<br><br>(min/day)	7±8	10±14	3±9<br><br>(data provided by author)	7±10	7±8	0±4<br><br>(data provided by author)
Cruz (2016)<br><br>(min/day)	32.2±15.4	57.8±32.8	25.6±25.4<br><br>(correlation coefficient input)	26.6±14.6	26.7±19.6	0.1±14.8<br><br>(correlation coefficient input)
Geidl (2021)	28.5±22.0	36.5±25.0	8±19.6<br><br>(correlation coefficient input)	30.4±24.2	34.7±26.9	4.3±21.2<br><br>(correlation coefficient input)
Louvaris (2016)<br><br>(min/day)	13±15	20±19	7±16<br><br>(data provided by author)	12±19	12±17	0±19<br><br>(data provided by author)
O'Neill (2018)<br><br>(min/day)	14.3±15.3<br><br>10+ time: 0.87±2.0	24.49±26.0<br><br>10+ time: 11.67±21.5		13.9±15.2<br><br>10+ time: 0.98±2.5	12.8±20.0<br><br>10+ time: 0.1±0.4	
Vasilopoulou (2017)<br><br>(min/day)			Group A: 4±2<br><br>Group B: 6±3<br><br>Combined: 5±2.7			−3±3<br><br>(data provided by author)

			(data provided by author, groups combined within RevMan)			
**SenseWear**						
Burge (2021)<br><br>(min)	46±61	49±55		48±65	50±56	
Burtin (2015)<br><br>(min/day)<br><br>*(median [IQR])*	17 (4-50)		Not reported in text	12 (2-26)		Not reported in text
Chaplin (2022)	82.03±69.9	65.7±39.7	−16.4±72.9<br><br>(raw data provided by author)	102.9±78.5	110±69.4<br><br>(raw data provided by author)	8.3±76.8<br><br>(raw data provided by author)
Deering (2016)	PR+A = 57.9±97.5<br><br>PR = 28.3±36.2		PR+A = −34.1±81.7<br><br>PR = −2.8±38.2	49.2±134.1		3.2±11
Holland (2017)<br><br>*Baseline: median (IQR)*<br><br>*Change scores: mean (95% CI)*	68 (29-121)		11.37 (−8.27 to 31.01)<br><br>±47.6 (SD calculated from 95% CI)	79 (24-136)		5.12 (−12.00 to 22.25)<br><br>±48.3 (SD calculated from 95% CI)
Horton (2021)<br><br>(min/day)<br><br>*Change scores: mean (95% CI)*	66±86<br><br>(data provided by authors)	84±64<br><br>(data provided by authors)	18 (−5 to 42)<br><br>±57 (SD calculated from 95% CI)	58±36<br><br>(data provided by authors)	50±39<br><br>(data provided by authors)	−8 (−28 to 73)<br><br>±122 (SD calculated from 95% CI)
Lahham (2020)<br><br>*Baseline: median (IQR)*	42 (24-80)		−5 (−301 to 290)	41 (14-112)		−211 (−497 to 76)

Change scores: mean (95% CI)						
Nolan (2017)<br>*(median [IQR])*	45 (20, 81)		11 (−1, 33)<br>14±29.1 (mean±SD calculated from median [IQR])	47 (18, 103)		11 (−2, 28)<br>14.7±30.6 (mean±SD calculated from median [IQR])
Pavitt (2020)<br>(min/day)<br>*(median [IQR])*	17 (8, 36)		2.0 (−3.6, 9.5)<br>2.4±41.8 (raw data provided by author)	43 (13, 94)		−7.0 (−30.0, 6.0)<br>−12.0±36.3 (raw data provided by author)
**GeneActiv**						
Cox (2022)<br>*Baseline: median (IQR)*<br>*Change scores: mean (95% CI)*	All CRD: 63 (29 to 111)		All CRD: 6.7 (−4.7 to 17.9)<br>COPD subgroup: −1 (−8 to 7)<br>±27.2 (SD calculated from 95% CI)	All CRD: 63 (38 to 99)		All CRD: 5.0 (−8.9 to 18.9)<br>COPD subgroup: −3 (−14 to 7)<br>±34.8 (SD calculated from 95% CI)
**Percentage of MVPA (% of wear time)**						
Park (2020)	3±2	5±3		4±2	4±3	
**Recommended MVPA (min/day)**						
Cruz (2016)	5.2±7.5	23.3±28.6		5.3±8.2	4.3±7.3	

**Time in light PA (min/day)**						
**ActiGraph**						
Armstrong (2021)<br><br>(min/day)	167±56	187±73		135±57	133±48	
Louvaris (2016)<br><br>(min/day)	135±62	160±67		144±56	137±65	
Vasilopoulou (2017)<br><br>(min/day)			Group A: 38±12<br><br>Group B: 28±11			−12±6
**SenseWear**						
Burge (2021)<br><br>(min)	191±98	178±74		217±129	214±106	
Horton (2021)<br><br>(min/day)	112±59<br><br>(data provided by authors)	136±73<br><br>(data provided by authors)	24 (−10 to 45)	107±72<br><br>(data provided by authors)	113±73<br><br>(data provided by authors)	6 (−23 to 33)
**GeneActiv**						
Cox (2022)	All CRD: 271±106		All CRD: −4.1 (−35.6 to 27.4)<br><br>COPD subgroup: −14 (−53 to 26)	All CRD: 285±90		All CRD: 9.5 (−21.2 to 40.2)<br><br>COPD subgroup: 0.5 (−33 to 34)
**Percentage light PA (% of wear time)**						
Park (2020)	18±9	21±8		20±6	19±6	
**Time in lifestyle PA (min/day**						
Louvaris (2016)<br><br>(min/day)	39±24	48±32		38±26	34±25	
Vasilopoulou (2017)<br><br>(min/day)			Group A: 11±4<br><br>Group B: 11±5			−7±4

**Time in moderate PA (min/day)**						
**SenseWear**						
Perez-Bogerd (2018)<br><br>*(geometric mean [geometric interval])*	36 (25, 52)	3 months: 37 (26, 54)<br><br>6 months: 33 (23, 48)		54 (38, 78)	3 months: 44 (31, 64)<br><br>6 months: 41 (29,60)	
**Percentage of moderate activity (%)**						
Chaplin (2022)	10.25±8.8	9.43±4.2		8.07±5.9	8.14±7.3	
**Time in vigorous PA (min/day)**						
Horton (2021)<br><br>(min/day)	2±5<br><br>(data from author)	6±21<br><br>(data from author)	4 (−4 to 14)	6±25<br><br>(data from author)	2±7<br><br>(data from author)	−4 (−15 to 7)
**Total PA (min/day)**						
Burtin (2015) (at least 2.0 METs)<br><br>(min/day)	40 (17-109)		Not reported in text	34 (17-69)		Not reported in text
Cruz (2016)	235.4±44.6	279.5±74.0		208.4±78.9	212.0±53.9	
Hansen (2020) (active time)	196±121		−29.0 (−95.4 to 29.9)	276±134		−38.3 (−107.3 to 21.7)
Wallaert (2020) (time in EE >2.5 METs)	149±125	137±75.4		151.9±122.4		
**Daily PA (steps + METs)**						
Altenburg (2015)<br><br>*(median [IQR])*	Total: 6563 (3919-8847)<br><br>Primary: 8043 (6111-10,567)<br><br>Secondary: 5816 (3863-8880)	Total: 8239 (5453-10207)<br><br>Primary: 8857 (7703-10747)		Total: 6238 (4530-8986)<br><br>Primary: 6202 (3583-9205)<br><br>Secondary: 5536 (4490-9315)	Total: 5592 (3967-8911)<br><br>Primary: 5328 (3572-9820)<br><br>Secondary: 5726 (4087-8626)<br><br>PR: 5389 (3967-9322)	

	PR+PA: 5110 (3291-7579)	Secondary: 8685 (5453-12674)<br><br>PR+PA: 6423 (4735-8414)		PR: 7371 (4951-8835)		
**Total PA level (MET/mins/week)**						
O'Neill (2018)	1464.1±1553.3	2427.7±1559.7		1797.5±1693.0	2229.9±2189.9	
**Self-reported minutes of activity (minutes)**						
Kesten (2008)<br><br>*(mean [SE])*	279 (41)	403 (81)	145 (84)	253 (50)	353 (100)	66 (96)
**Average movement intensity (vector magnitude counts/min)**						
**ActiGraph**						
Armstrong (2021)	337±154	410±231		307±170	287±133	
Cerdan-de-las-Heras (2021a)	480.3±115	444±180		412.5±178	393±186	
Cerdan-de-las-Heras (2021b)	294.4±131.2	303.7±133.5		420.2±213.2	420.4±282.7	
Cerdan-de-las-Heras (2022)	561.7±242.9	537.1±160.5		490.1±240.5	423.0±162.4	
Louvaris (2016)	411±190	495±213		401±184	406±205	
Park (2020)<br><br>(total activity count/wear time)	215.64±103.16	275.09±99.79		258.85±105.73	258.59±111.47	
**Ambulatory activity monitor**						
Sewell (2005)	Not reported		40.63% (7.42 to 73.83%) (% improvement)	Not reported		29.18% (3.19 to 55.17%) (% improvement)

(“activity monitor counts”)						
**Movement intensity (m/s²)**						
Breyer (2010)	1.59±0.47		0.40±1.4	1.50±0.29		
Troosters (2018)<br>*(mean±SE)*	SMBM + placebo: 1.91±0.02		SMBM + placebo: 0.039±0.029	Not reported		SMBM + tiotropium: 0.049±0.029<br>SMBM + tiotropium/ olodaterol: 0.098±0.029<br>SMBM + tiotropium/ olodaterol + exercise: 0.000±0.029
**Total energy expenditure (kcal/day)**						
Deering (2016)	PR+A = 2214±563.3<br>PR = 2014±543		PR+A = −203.3±539.8<br>PR = −45.9±222.2	2026±462.5		−8.0±92.9.5
Holland (2017)	Not Reported		−33 (−547 to 482)	Not Reported		−294 (−746 to 157)
Lahham (2020)	9363±2572		−4 (−1425 to 1418)	9953±2458		82 (−1299 to 1463)
Rausch Osthoff (2021)	2324±746	2196±833		2440±472	2208±416	
Wallaert (2020)	2343±268	2369±390		2630±742	Not Reported	
**Estimated daily energy expenditure**						
Felcar (2018)	219.2±126.2	3 months: 258.5±127.8<br>6 months: 289.8±154.0		210.7±115.5	3 months: 232.7±113.3<br>6 months: 242.9±116.9	

**Active energy expenditure METs (kcal/day)**						
Horton (2021) (>3 METs)	347±452<br><br>(data provided by author)	430±323<br><br>(data provided by author)	83 (−124 to 288)	330±366<br><br>(data provided by author)	236±186<br><br>(data provided by author)	−94 (−61 to 63)
Wallaert (2020) (>2.5 METs)	572±410	571±256		703±639		
**Daily metabolic equivalence to tasks (METs)**						
Cedeño de Jesús (2022)	1.42±0.35	1.57±0.35		1.4±0.3		1.26±0.36
Chaplin (2022)	1.52±0.3	1.54±0.2		1.44±0.4	1.42±0.4	
Deering (2016)	PR+A = 1.3±0.4<br><br>PR = 1.2±0.2		PR+A = −0.0±0.2<br><br>PR = 0.01±0.2	1.2±0.23		0.0±0.1
Holland (2017)	Not Reported		0.03 (−0.04 to 0.09)	Not Reported		−0.01 (−0.06 to 0.05)
Lahham (2020)	1.2 (1.0-1.4)		0.1 (−0.1 to 0.2)	1.1 (1.0-1.3)		0.0 (−0.2 to 0.2)
Rausch Osthoff (2021)	1.3±0.3	1.3±0.3		1.5±0.5	1.4±0.2	
**Total METs of PA a week**						
Rausch Osthoff (2021)	4246±4715	8819±9632		2437±2731	6400±5288	
Gaunuard (2014)<br><br>(area under the curve; cumulative MET/mins, reported weekly for 3 months)			0-3 months: 51364±57713			0-3 months: 20892±37155
**Number of bouts of PA**						
Chaplin (2022)	2-min bouts: 21.1±18.5<br><br>5-min bouts: 7.2±8.5	2-min bouts: 21.4±8.9<br><br>5-min bouts: 6.7±4.0		2-min bouts: 18.0±12.7<br><br>5-min bouts: 5.5±4.8	2-min bouts: 17.9±14.6<br><br>5-min bouts: 5.9±6.0	

	10-min bouts: 2.11±2.8<br><br>20-min bouts: 0.47±0.7	10-min bouts: 2.1±1.8<br><br>20-min bouts: 0.43±0.4		10-min bouts: 1.6±2.1<br><br>20-min bouts: 0.26±0.4	10-min bouts: 1.6±1.9<br><br>20-min bouts: 0.38±0.5	
Holland (2017)<br><br>(MVPA bouts ≥ 10 mins)	0.67 (0.06-2.58)		0.30 (−0.22 to 0.82)	0.75 (0.13-2.63)		−0.25 (−0.20 to 0.71)
Lahham (2020))<br><br>(MVPA bouts ≥ 10 mins)	1.0 (0.3-2.2)		−0.3 (−1.6 to 1.0)	0.7 (0.2-3.8)		−0.6 (−1.9 to 0.7)
O'Neill (2018)	10+: 0.05±0.1	10+: 0.57±1.1		10+: 0.06±0.2	10+: 0.01±0.04	
**Mean bout length (minutes)**						
Chaplin (2022)	2.8±0.8	2.7±0.7		2.7±0.9	2.8±1.0	
Holland (2017)<br><br>(MVPA bouts; min/day)	11 (1-34)		16.49 (3.13 to 29.84)	12 (1-42)		5.11 (−6.69 to 16.91)
Horton (2021) (time in >3 METs in >10min bouts)<br><br>(min/day)	Not reported in text		28 (9 to 48)	Not reported in text		−4 (−13 to 6)
Lahham (2020)<br><br>(MVPA bouts; min/day)	20 (5-43)		−4 (−29 to 22)	9 (2-63)		−13 (−38 to 12)
**Daily walking time (minutes/day)**						
Breyer (2010)	46.7±35.2		14.9±1.9	42.3±36.5		Not reported in text
Burtin (2015)	33 (16-47)		Not reported in text	29 (17-44)		Not reported in text
Kawagoshi (2015)	Not reported in text		51.3±63.7	Not reported in text		12.3±25.5

Troosters (2018)<br><br>*(mean±SE)*	SMBM + placebo: 65.17±31.82		SMBM + placebo: 9.56±3.49	SMBM + tiotropium: 67.28±31.24<br>SMBM + tiotropium/ olodaterol: 70.76±39.55<br>SMBM + tiotropium/ olodaterol + exercise: 68.02±31.37		SMBM + tiotropium: 6.91±3.49<br>SMBM + tiotropium/ olodaterol: 10.66±3.43<br>SMBM + tiotropium/ olodaterol + exercise: 6.31±3.37
**Self-reported walking (min/day)/(days/week)**						
Cameron-Tucker (2016)	Mins/day: 10±30<br><br>Days/week: 3±5		Mins/day:<br>TP1/2: 14±26<br><br>TP2/3: 16±23<br><br>Days/week:<br>TP1/2: 0±5<br><br>TP2/3: 0±1	Mins/day: 3±23<br><br>Days/week: 0±4		Mins/day:<br>TP1/2: 16±40<br><br>TP2/3: 17±29<br><br>Days/week:<br>TP1/2: 0±0<br><br>TP2/3: 0±1
**Frequency of postural changes (times/day)**						
Kawagoshi (2015)	Not reported in text		Total: 40±66<br><br>Getting-up: 0.1±25<br><br>Standing-up: 43±60	Not reported in text		Total: 19±44<br><br>Getting-up: 6±31<br><br>Standing-up: 14±34
**Time with different MET intensities**						
Rausch Osthoff (2021)	Low MET: 1321±130<br><br>Medium MET: 64±83<br><br>High MET: 2±4<br><br>Very high MET: 0.2±1.1	Low MET: 1275±183<br><br>Medium MET: 53±44<br><br>High MET: 1±3<br><br>Very high MET: 0±0		Low MET: 1299±137<br><br>Medium MET: 78±62<br><br>High MET: 19±33<br><br>Very high MET: 1.7±3.7	Low MET: 1329±75<br><br>Medium MET: 57±48<br><br>High MET: 3±6<br><br>Very high MET: 0.1±0.3	

**PAL per day (total energy expenditure / sleep energy expenditure)**						
Curtis (2016)	1.49±0.19		−0.06±0.16	1.39±0.20		0.04±0.15
Pavitt (2020)	3.0 (2.4, 4.3)	0.0 (0.0, 0.0)		3.0 (2.6, 3.8)	0.0 (−1.0, 1.0)	
Rausch Osthoff (2021)	1.6±0.3	1.5±0.3		1.6±0.3	1.5±0.1	
**Time spent standing (min/day)**						
Breyer (2010)	215±182		129±26	222±169		Not reported in text
Kawagoshi (2015)	Not reported in text		43.0±28.1	Not reported in text		31.3±46.8
**Clinical PROactive Physical Activity in COPD (C-PPAC) scores**						
Armstrong (2021)	Total: 60±16<br>Difficulty: 62±15<br>Amount: 58±20	Total: 69±16<br>Difficulty: 69±15<br>Amount: 69±20		Total: 59±14<br>Difficulty: 62±16<br>Amount: 56±19	Total: 60±15<br>Difficulty: 61±15<br>Amount: 56±19	
**Daily version of C-PPAC (D-PPAC) total score**						
Troosters (2018)<br>*(mean±SE)*	SMBM + placebo: 62.20±0.66		SMBM + placebo: 3.10±0.90	Not reported		SMBM + tiotropium: 5.13±0.94<br>SMBM + tiotropium/ olodaterol: 5.48±0.93<br>SMBM + tiotropium/ olodaterol + exercise: 5.19±0.90
**Baecke scores**						
Varas (2018)	Total: 6.5±3.2<br>Domestic: 1.3±0.8<br>Sport: 3.9±2.9<br>Leisure: 2.3±1.5	Total: 1.7±2.2<br>Domestic: 0.1±0.3<br>Sport: 0.5±1.1<br>Leisure: 0±0.7		Total: 4.5±2.8<br>Domestic: 1.7±0.6<br>Sport: 1.7±2.6<br>Leisure: 1.1±0.6	Total: 0±1.4<br>Domestic: 0±0.3<br>Sport: 0.1±1.2<br>Leisure: 0±0.5	

**Smoking, Nutrition, Alcohol consumption, Physical activity, Psychological well-being, and Symptom management (SNAPPS) PA score**						
Cameron-Tucker (2016)	5±8		TP1/2: 0±4<br><br>TP2/3: 0±1	0±8		TP1/2: 0±4<br><br>TP2/3: 0±0
**Community Healthy Activities Model Programme for Seniors (CHAMPS)**						
Bentley (2020)	Completed PR: 2017.3 (1220.5-5237.3)<br><br>Withdrawn: 2468.5 (1193.8-3138.7)	Not reported		Not reported	Not reported	

Supplementary material D – Details of intervention and comparator groups for individual studies

First author (Year)	Experimental group					Comparison group(s)				
	**Intervention type**	**Sample size (n)**	**Mode of delivery**	**Frequency**	**Duration**	**Intervention type**	**Sample size (n)**	**Mode of delivery**	**Frequency**	**Duration**
Aldhahir (2021)	**PR + protein supplementation:**<br><br>*PR:*<br><br>Delivered according to BTS guidelines<br><br>*Nutrition supplementation:*<br><br>125 ml of Fortisip Compact Protein (Nutricia, Zoetermeer, Netherlands). 300 kcal, 24% protein, 41% carbohydrate, 35% fat	36 (22 completed)	Centre-based	*PR:* Twice weekly<br><br>*Nutrition supplement:* 2 bottles/day	6 weeks	**PR + placebo supplementation**<br><br>*PR:*<br><br>Same as in experimental<br><br>*Placebo supplement:*<br><br>200 ml of PreOp (Nutricia). 100 kcal and 100% carbohydrate.	32 (22 completed)	Centre-based	*PR:* Twice weekly<br><br>*Placebo supplement:* 2 bottles/day	6 weeks
Altenburg (2015)	**PR + PA counselling:**<br><br>*PR:*<br><br>Exercise training (cycling, walking, swimming and	31	Centre-based	*PR:* 3 sessions /week<br><br>*PA counselling:* 5 sessions	*PR:* 9 weeks<br><br>*PA counselling:* 12 weeks	**PR:**<br><br>Same as in experimental<br><br>OR	30<br><br>24	Centre-based	3 sessions/ week	*Usual care:* 12 weeks,<br><br>*PR:* 9 weeks

	sports) and educational courses with psychological and/or nutritional support if necessary<br><br>*PA counselling:*<br><br>based on goal-setting and implementation of goals, pedometer feedback and motivation, step/activity diary			over 3 months		**Usual care** (from primary care)<br><br>**Usual care** (from secondary care)<br><br>*(Usual care from primary and secondary care combined in meta-analyses)*	23			
Armstrong (2021)	**PR + PA modification:**<br><br>*PR:*<br><br>Delivered according to BTS guidelines<br><br>*PA modification:*<br><br>Motivational interview, provision of a pedometer, individualised step-count target, and a step-count diary	24	*PR*: centre-based	Twice weekly	8 weeks	**PR:**<br><br>Delivered according to BTS guidelines	24	Centre-based	Twice weekly:<br><br>2 x 60-min exercise sessions/wee k<br><br>1 x 30-min education session/week	8 weeks
Bentley (2020)	**PR + PA promotion:**<br><br>*PR:*	19 (10 completed)	*PR:* centre-based	Twice weekly	8 weeks	**PR:**<br><br>Same as in experimental	11 (6 completed)	Centre-based	Twice weekly	6-7 weeks

	Physical exercises of upper and lower extremities and education sessions<br><br>*PA promotion (mobile health):*<br><br>Self-Management supported by Assistive, Rehabilitative, and Telehealth technologies-COPD (SMART-COPD)		*Mobile health:* delivered via a smart-phone app and activity tracker							
Breyer (2010)	**PR + Nordic walking:**<br><br>Participants received a 2-hour instruction session on Nordic walking. Goal of achieving 75% initial maximum heart rate.<br><br>Received one educational session (including pulmonary pathophysiology, management of breathlessness and exacerbations).	32 (30 completed)	Outdoors, supervised	3 sessions/ week	3 months	**Usual care**<br><br>All participants received one education session/week	33 (30 completed)	Centre-based	Once weekly	3 months

Burge (2021)	**PR:**<br><br>One home visit (established exercise goals, create formal exercise prescription, provide education in use of home diary, assess inhaler technique) then weekly telephone calls	33 (25 completed)	Home-based	"At least 30 min aerobic training most days of the week"	8 weeks	**PR:**<br><br>Individually prescribed exercise training and self-management education. 30-min aerobic training plus resistance exercises	40 (36 completed)	Centre-based	Twice weekly	8 weeks
Burtin (2015)	**PR + PA counselling:**<br><br>*PR:*<br><br>Exercise and education components.<br><br>*PA counselling:*<br><br>Evaluated motivation to change PA behaviour. Counselling focused on action planning, goal setting, facilitating barrier identification, and relapse prevention when motivation to change scores were high and	40 (30 completed)	Centre-based	PR: 3 sessions/ week (first 3 months), 2 sessions/wee k (second 3 month period)<br><br>PA counselling: 8 individual sessions (20-30 mins) over course of intervention	6 months	**PR:**<br><br>*PR:*<br><br>Exercise and education components<br><br>*Sham attention:*<br><br>Intermediate evaluation of PA but no structured feedback provided	40 (31 completed)	Centre-based	*PR:* 3 sessions/ week (first 3 months), 2 sessions/ week (second 3 month period)<br><br>*Sham attention:* similar duration and timing as individual-ised sessions within experimental group	6 months

	communicative approaches based on motivational interviewing to enhance motivation when motivation to change was low.									
Cameron-Tucker (2016)	**Tele-rehabilitation:** Completed a summary of their SNAPPS health behaviours and established a home-based walking plan, aiming to meet Australian guidelines to walk at a moderate intensity, to accumulate 30 minutes daily on several and preferably all days of the week. They received a copy of their written personal walking action plan, their personal SNAPPS summary, plus information concerning health behaviours	35	Home-based	Daily	8 weeks	**Usual care**	30	N/A	N/A	8 weeks

Camillo (2020)	**PR with downhill walking therapy:**<br><br>*PR:*<br><br>Cycling, walking (up to 20 mins), upper and lower limb strength training, arm cranking and stair climbing.<br><br>*Downhill walking:*<br><br>performed at a fixed −10% inclination (i.e. a 10 m decline for every 100 m walked) via insertion of a customised bracket underneath the treadmill, secured against the rear feet	24 (18 analysed)	Centre-based	3 sessions/ week	12 weeks	**PR with conventional walking therapy:**<br><br>*PR:*<br><br>Same as experimental group but involving conventional walking on a motorised treadmill with neutral inclination, progressed via increases in duration, speed and inclination (positive)	20 (17 analysed)	Centre-based	3 sessions/ week	12 weeks
Cedeño de Jesús (2022)	**PR:**<br><br>Included educational measures and learning of self-management techniques. Two initial centre-based sessions were	18 (16 analysed)	Home-based	3-5 sessions/ week	8 weeks	**PA promotion:**<br><br>Participants received written instructions on how to perform PA and progressively increased walking	16 (15 analysed)	Home-based	3-5 sessions/ week	8 weeks

	performed including an educational workshop (also explained exercises to perform at home) and supervised exercise session.<br><br>During home-based PR, motivational calls were carried out weekly. Participants were given a diary for daily PA collection. Participants performed both strength (upper and lower limb) and endurance (walking or cycling) training.					intensity. Recommended to perform it at least 3-5 times/week for 30 min. They did not receive supervision.				
Cerdan-de-las-Heras (2021a)	**Tele-rehabilitation:**<br><br>Delivered using a virtual autonomous physiotherapist agent (VAPA) platform.<br><br>VAPA consists of (a) multidimensional	15	Home-based	3-5 sessions/ week	12 weeks	**Usual care**	14	N/A	N/A	12 weeks

	software serving as a service platform for therapists to create customised tele-rehab programmes and (b) a mobile app for patients to install connected to biometric sensor attachable to patient's chest, arms, or fingers to collect oximetry and heart rate data, enabling rehab programme to adjust in real time.									
Cerdan-de-las-Heras (2021b)	**Tele-rehabilitation:**<br><br>As in Cerdan-de-las-Heras (2021a)	27	Home-based	3-5 sessions/ week	8 weeks	**PR:**<br><br>Conventional standardised rehab programme<br><br>6 hours of education about COPD and its treatment	27	Centre-based	Twice weekly	8 weeks
Cerdan-de-las-Heras (2022)	**Tele-rehabilitation:**<br><br>As in Cerdan-de-las-Heras (2021a)	15	Home-based	3-5 sessions/ week	12 weeks	**Usual care**	15	N/A	N/A	12 weeks
Chaplin (2022)	**Web-based PR:**<br><br>Involves home exercise	51 (22 completed ; 20 analysed	Home-based	Daily	7 weeks	**PR:**<br><br>Conventional PR delivered	52 (40 completers ; 34 analysed	Centre-based	Twice weekly	7 weeks

	programme and goal setting as well as a personalised action plan and education content.<br><br>Patients allowed to work through website content at their own pace, however, certain milestones need to be completed/ achieved before further content can be accessed to ensure appropriate progress.	for PA data)				according to BTS guidelines	for PA data)			
Cox (2022)	**Tele-rehabilitation:**<br><br>Participants were provided necessary equipment (including exercise bike and tablet computer). Initial exercise training session undertaken during home-visit with physiotherapist. Remaining sessions conducted in virtual group of up to 6 participants.<br><br>Consisted of both exercise	71 (68 analysed; 47 COPD analysed)	Home-based	Twice weekly (supervised)	8 weeks	**PR:**<br><br>Consisted of endurance (walking and cycling) and resistance (upper and lower limb) exercises.<br><br>Prescribed a home walking programme and encouraged to performed an additional 3 unsupervised sessions per week.	71 (67 analysed; 49 COPD analysed)	Centre-based	Twice weekly (supervised)	8 weeks

	(endurance [cycling] and resistance [using available household items] training), and education sessions. Participants were also prescribed a home walking programme and encouraged to performed an additional 3 unsupervised sessions per week.<br><br>Education was provided via resources (printed book and brochure) and available in group format (virtual).					Education was provided via resources (printed book and brochure) and available in group format (centre-based).				
Cruz (2016)	**PR + PA focused behavioural intervention:**<br><br>*PR:*<br><br>Exercise training (aerobic, resistance, and balance training) and psychosocial support and education sessions.	16 (13 analysed)	Centre-based	3 sessions/ week	12 weeks	**PR:**<br><br>Same as experimental group	16 (13 analysed)	Centre-based	3 sessions/ week	12 weeks

	PA focused behavioural intervention:<br><br>Received pedometer, individualised short- and long-term step-count goals, and step-count calendar									
Curtis (2016)	**PR + angiotensin-converting enzyme inhibition (ACE-I):**<br><br>*PR:*<br><br>Delivered according to ATS/ERS/BTS guidelines<br><br>*ACE-I:*<br><br>10 mg enalapril once daily	39 (31 completed)	Centre-based with 1 home-based session	3 sessions/ week	*PR:* 8 weeks<br><br>*ACE-I:* 10 weeks	**PR + placebo**<br><br>*PR:*<br><br>Same as experimental<br><br>*Placebo:*<br><br>microcrystalline cellulose	39 (34 completed)	Centre-based with 1 home-based session	3 sessions/ week	PR: 8 weeks<br><br>Placebo: 10 weeks
de Blok (2006)	**PR + PA counselling:**<br><br>*PR:*<br><br>According to ACCP/AACVPR guidelines<br><br>*PA counselling:*	10 (8 analysed)	Centre-based	*PA counselling:* 4 sessions<br><br>*PR:* unclear	9 weeks	**PR:**<br><br>Same as experimental group	11 (8 analysed)	Centre-based	Unclear	9 weeks

	Received 4 exercise counselling sessions (focusing on motivation, goal-setting, shifting boundaries, and consolidation of PA behaviour). Pedometer used as a motivational and feedback tool.									
Deering (2011)	**PR+acupuncture**<br><br>*PR:*<br><br>Exercise and education sessions based on ATS/ERS guidelines. Inspiratory muscle training (5 times/week)<br><br>*Acupuncture:*<br><br>20 mins/week by physiotherapist trained in traditional Chinese medicine on the forearm	16	Centre-based	*PR:*<br><br>Twice weekly (with recommend-ation for 3 additional unsupervised home-based sessions)<br><br>*Acupuncture*:<br><br>Once weekly	**PR:** 7 weeks<br><br>*Acupuncture*: 7 weeks	**PR:**<br><br>Same as experimental<br><br>**Usual care**	*PR:* 25<br><br>*Usual care:* 19	*PR:* Centre-based<br><br>*Usual care:* N/A	*PR:* Twice weekly (with recommend-ation for 3 additional unsupervised home-based sessions)<br><br>*Usual care:* N/A	*PR:* 7 weeks<br><br>*Usual care:* 7 weeks
Duiverman (2008)	**PR + Non-invasive positive pressure ventilation (NIPPV)**	37 (31 baseline, 24 analysed)	Centre-based	*PR:* 3 sessions/wee k<br><br>*NIPPV:* daily	3 months	**PR:**<br><br>Same as experimental group	35 (32 analysed)	Centre-based	3 sessions/wee k	12 weeks

	PR:<br><br>Consisted of strength training, cycling, walking, inspiratory muscle training, education and psychological and/or nutritional support.<br><br>*NIPPV:*<br><br>Instituted on nocturnal bilevel NIPPV in the hospital immediately before starting PR.									
Effing (2011)	**PR:**<br><br>"Community-based physiotherapeutic exercise programme (COPE-active)" consisting of endurance or interval training as well as resistance training. Included bicycling, walking, climbing stairs and lifting weights.	80 (77 baseline; 74 follow-up)	Centre- and home-based	*First 6 months (compulsory)*: centre-based (2 times/week), home-based (once weekly)<br><br>*Final 5 months (recommended):* centre-based (once weekly), home-based (once weekly)	11 months (6 months compulsory, 5 months recommended)	**Self-management:**<br><br>2-hour small-group sessions with the intention to change disease behaviour by increasing knowledge, confronting them with consequences of specific behaviour, and helping them acquire and practice self-	79 (76 baseline, 68 follow-up)	Centre-based	Four weekly sessions	Unclear

	Education/self-management sessions as in comparison group.					management skills.<br><br>Supplied with a booklet with content of educations sessions.				
Felcar (2018)	**Water-based PR:**<br><br>Training sessions involved a warm up, endurance training (pool cycling and walking dictated by speed achieved during six-minute walk test), strength training (pool-based upper and lower limb weights), and stretching.<br><br>Educational sessions offered every 2 weeks.	34 (20 analysed)	Centre-based	3 sessions/ week (first 3 months)<br><br>Twice weekly (last 3 months)	6 months	**PR:**<br><br>*Land-based:*<br><br>As in experimental group but on land.<br><br>Educational sessions offered every 2 weeks.	36 (16 analysed)	Centre-based	3 sessions/ week (first 3 months)<br><br>Twice weekly (last 3 months)	6 months
Gaunaurd (2014)	**PR:**<br><br>Included educational lectures and supervised aerobic and strengthening exercises.	14 (11 analysed)	Centre-based	Twice weekly	12 weeks	Usual care	11 (10 analysed)	N/A	N/A	12 weeks
Geidl (2021)	**PR + pedometer-based PA promotion**	167	Centre-based	PR: Unclear	3 weeks	**PR:**	160	Centre-based	Unclear	3 weeks

	PR:<br><br>consisted of physical exercise (endurance, strength, and whole-body vibration training), COPD patient education, and respiratory physiotherapy.<br><br>*PA promotion:*<br><br>pedometer-based, two 45-minute sessions focused on behaviour change (goal setting, self-monitoring, feedback), received booklet for recording step count.			PA promotion: two 45-min sessions		As in experimental group				
Hansen (2020)	**Tele-rehabilitation:**<br><br>Standardised via a video conference software system installed on a single touch screen. Exercise (high-repetitive time-based muscle	67 (57 completed)	Home-based	3 sessions/ week	10 weeks	**PR:**<br><br>Followed Danish Health Authority's National Clinical Guideline and Regional Guidelines. Exercise (endurance and	67 (43 completed)	Centre-based	Twice weekly	10 weeks

	endurance training) and education sessions.					resistance training) and education sessions.				
Holland (2017)	**Home-based PR:**<br><br>As in Burge (2021)	80 (73 completed)	Home-based	"At least 30 min aerobic training most days of the week"	8 weeks	**Centre-based PR:**<br><br>As in Burge (2021)	86 (42 completed)	Centre-based	Twice weekly	8 weeks
Horton (2021)	**Home-based PR:**<br><br>Supported by SPACE for COPD manual. Initial introductory session led by a healthcare professional trained in motivational interviewing. Received two telephone calls at weeks 2 and 4 to assess progress, support and motivation.	26	Home-based	Not reported	7 weeks	**Centre-based PR:**<br><br>Exercise and education programme.	25	Centre-based	Twice weekly	7 weeks
Jarosch (2020)	**PR:**<br><br>Consisted of medical care (as required), psychological support, breathing therapy, education (disease management, physical activity, nutritional	36 (34 analysed)	Centre-based	5-6 sessions/ week	3 weeks	**Usual care**	18 (17 analysed)	N/A	N/A	3 weeks

	counselling, motivation) and an exercise training program (endurance of interval cycling) and resistance training (major muscle groups).									
José (2021)	**PR:**<br><br>Home-based, non-supervised sessions of aerobic [stepping exercise] and resistance [elastic band exercises] training	33 (27 completed)	Home-based	3 sessions/ week	8 weeks	**Education booklet:**<br><br>Containing instructions regarding how to perform the physical activities and walking at moderate intensity, to be performed three times per week for 30 min. They did not receive any supervised physical training.	30 (28 completed)	Home-based	3 times/week	8 weeks
Kawagoshi (2015)	**PR + pedometer feedback:**<br><br>*PR:*<br><br>Breathing retraining, exercise training and monthly 45-min education	12	Home-based	"Patients were instructed to practice this program daily at home"	1 year	**PR:**<br><br>Same as experimental group	15	Home-based	"Patients were instructed to practice this program daily at home"	1 year

	programme<br><br>*Pedometer feedback:*<br><br>Administered with pedometer and received monthly feedback about pedometer use/average daily PA from previous month for 1 year									
Kesten (2008)	**PR + tiotropium:**<br><br>*PR:*<br><br>Consisted of treadmill training for at least 30 minutes/session. Also involved upper limb activities and education.<br><br>*Medication:*<br><br>Tiotropium (18 µg) once daily for 5 weeks prior to, 8 weeks during, and 12 weeks following PR.	55	Centre-based	3 sessions/ week	8 weeks	**PR:**<br><br>Same as experimental group.	53	Centre-based	3 sessions/ week	8 weeks
Lahham (2020)	**Home-based PR:**	29	Home-based	"Encouraged to work towards	8 weeks	**Usual care:**	29	N/A	N/A	8 weeks

	Endurance [walking] and resistance [upper and lower limb] training. Weekly phone calls - principles of motivational interviewing, encouraged participants to identify own motivation and set goals.			achieving a goal of participating in 30 mins whole-body exercise training 5 days/week"		Advised to keep active but no specific exercise advice was provided. Weekly phones to control for attention.				
Louvaris (2016)	**PR including interval cycling training:**<br><br>Interval training (cycled at a mean intensity of 130% of baseline peak work rate for 45 mins [30s exercise, 30s rest]), resistance training (upper and lower limbs at 70% one repetition maximum), breathing retraining, dietary advice and education sessions.	85	Centre-based	3 sessions/ week	12 weeks	**Usual care**	43	N/A	N/A	12 weeks
Nolan (2017) /	**PR + pedometer step targets:**	76 (63 analysed)	Centre-based	Twice weekly	8 weeks	**PR:**	76 (59 analysed)	Centre-based	Twice weekly	8 weeks

Polgar (2021)	*PR:*<br><br>Delivered according to BTS guidelines on PR<br><br>*PA promotion:*<br><br>Administered with pedometer, individualised daily pedometer step-count target with weekly review for 8 weeks, step-count diary. Target was an increase of 5% on the preceding week's average step-count. Each patient counselled on the importance of achieving the step-count and given advice on how to increase PA levels					Delivered according to BTS guidelines on PR				
O'Neill (2018)	**PA intervention:**<br><br>Clinician-facilitated pedometer-driven walking programme. Provided with	23	Centre-based	Daily	12 weeks	**PR:**<br><br>Consisted of exercise (cardio-vascular exercises and lower and upper	26	Centre-based	Twice weekly	6 weeks

	pedometer and a manual with weekly step diary and action and coping plans. Weekly contact with trained physiotherapist or nurse					strengthening exercises)				
Park (2020)	**PR + app-based self-management:**<br><br>*PR:*<br><br>Involving group exercise (stretching and circuit training) and education sessions (including disease management, nutrition, smoking cessation etc).<br><br>*PA self-monitoring:*<br><br>Participants recorded time and type of exercise and step count from pedometer, as well as symptoms, bronchodilator use, and health care use due to exacerbations in smartphone app.	23 (22 completed)	Centre- and home-based	4 sessions/ week (first month)	6 months	**PR/Usual care**<br><br>*PR (during first month):*<br><br>Same as experimental group<br><br>**Usual care (during following 5 months)**	21 (20 completed)	Centre-based (first month)	4 sessions/ week (first month)	6 months

Pavitt (2020)	**PR + beetroot juice**<br><br>*PR:*<br><br>According to ATS/ERS guidelines. Mixture of aerobic and strength training.<br><br>*Nitrate-rich beetroot juice (BRJ):*<br><br>Commercially available concentrated BRJ cut with organic lemon juice (2%) and contained 0.8g of nitrate.	57	Centre-based	Twice weekly	8 weeks	**PR + placebo nitrate-deplete beetroot juice**<br><br>*PR:*<br><br>Same as in experimental group<br><br>*Placebo:*<br><br>Same beverage as experimental run through an ion exchange column, identical in taste.	65	Centre-based	Twice weekly	8 weeks
Perez-Bogerd (2018)	**PR:**<br><br>Exercise training and patient education/ occupational therapy/nutrition counselling/ psychological support	30 (22 3-month analysed; 20 6-month analysed)	Centre-based	3 sessions/ week for first 3 months then twice weekly for second 3 months	6 months	**Usual care**	30 (25 3-month analysed; 23 6-month analysed)	N/A	N/A	6 months
Polkey (2018)	**Tai chi:**<br><br>Participants were taught 2-3	60 (55 completed)	Centre-based	5 sessions/ week	12 weeks	**PR:**<br><br>Participants performed a	60 (55 completed)	Centre-based	3 sessions/ week	12 weeks

	movements each day and typically took 2 weeks to master them (supervised).<br><br>Thereafter, the participants were able to join larger group training by real-time video streaming.					mixture of resistance exercises (arm and leg), hybrid (rowing machine), and progressive aerobic whole body exercise (eg, cycle or treadmill) in addition to educational sessions.				
Rausch Osthoff (2021)	**PR + PA counselling:**<br><br>*PR:*<br><br>According to ATS/ERS guidelines<br><br>*PA counselling:*<br><br>5 face-to-face 30-min counselling sessions which included motivational interviewing techniques provided by two experienced physiotherapists independent to the rehab program	17	Centre-based	*PR:*<br><br>3 sessions/ week (2 at outpatient clinic, 1 outdoor Nordic walking training)<br><br>*PA counselling:*<br><br>5 face-to-face 30-min sessions	12 weeks	**PR:**<br><br>Same as in experimental group	26 (25 analysed)	Centre-based	3 sessions/ week (2 at outpatient clinic, 1 outdoor Nordic walking training)	12 weeks

Selzler (2021)	**PR + "enhanced" education:**<br><br>*PR:*<br><br>Exercise according to ATS/ERS guidelines.<br><br>*Enhanced education sessions:*<br><br>Incorporated more self-management content, emphasising behaviour change through goal setting and self-efficacy enhancement. Delivered in an interactive format that encouraged reflection and personal application	108 (99 completed)	Centre-based, education sessions delivered via telehealth technology at rural sites	Twice weekly or 3 sessions/ week	16 sessions (either 2 session/week for 8 weeks or 3 sessions/wee k for 6 weeks)	**PR:**<br><br>Exercise according to ATS/ERS guidelines.<br><br>Lecture style education sessions of traditional PR.	99 (85 completed)	Centre-based, education sessions delivered via telehealth technology at rural sites	Twice weekly or 3 sessions/ week	16 sessions (either 2 session/ week for 8 weeks or 3 sessions/ week for 6 weeks)
Sewell (2005)	**Individually targeted exercise sessions:**<br><br>Participants only completed exercises that were based on those daily activities identified during the Canadian	90 (64 completed)	Centre-based, Also completed daily training walks at home. Participants in both groups given a list	Twice weekly	7 weeks	**General exercise programme:**<br><br>Conventional programme of strengthening exercises<br><br>Aerobic training programme within PR:	90 (59 completed)	Centre-based, Also completed daily training walks at home. Participants in both groups given a list	Twice weekly	7 weeks

	Occupational Performance Measure (COPM) interview. Exercises specifically aimed to address these functional problems (individualised).<br><br>Aerobic training programme within PR: walking at 85% predicted peak VO2 from ISWT.		of exercises to complete at home.			walking at 85% predicted peak VO_2 from ISWT.		of exercises to complete at home.		
Troosters (2018)	**Self-management behaviour modification (SMBM) + tiotropium/ olodaterol + exercise training**<br><br>*Exercise training:*<br><br>Involved aerobic (cycling or walking) and resistance (upper and lower limb) training.<br><br>*SMBM:*<br><br>Education as part of self-management behavioural	76 (70 analysed)	Centre-based	**PR:** 3 sessions/ week	**PR:** 8 weeks<br><br>**Medication:** 12 weeks	**SBMB:** as in experimental group (plus placebo)<br><br>**SBMB + tiotropium:** 5 µg<br><br>**SMBM + tiotropium/ olodaterol:** 5 µg each	75 (65 analysed)<br><br>76 (67) analysed)<br><br>76 (72 analysed)	Centre-based	Self-management: at week 1, 2, 5, 8, and 11, as well as 15 (after follow-up)	12 weeks

	modification (adapted from "Living Well with COPD" programme).<br><br>*Medication:*<br><br>both tiotropium and olodaterol (5 µg).									
Van de Bool (2017)	**PR + nutrition supplementation**<br><br>*PR:*<br><br>According to ATS/ERS guidelines including high intensity endurance exercise by cycle ergometry and treadmill walking and progressive resistance exercise of upper and lower body at 75% of the 1 repetition maximum plus education sessions.<br><br>*Nutritional supplementation:*<br><br>Consumed 2-3 portions of	42 (38 completed)	Centre-based	2-3 sessions/ week	4 months (40 training sessions)	**PR + placebo supplementation**<br><br>*PR:*<br><br>Same as experimental group.<br><br>*Placebo:*<br><br>Consumed 2-3 portions of supplement per day (flavoured non-caloric aqueous solution)	39 (35 completed)	Centre-based	2-3 sessions/ week	4 months (40 training sessions)

	supplement per day (125ml, 187.5kcal [9.4g protein, 28.1g carbohydrate, 4.1g fat = 20%EN protein, 60EN% carbohydrate, 20EN% fat], enriched with leucine, n-3 PUFA and vitamin D)									
Varas (2018)	**PR + pedometer feedback:**<br><br>Walking 5 days/week for 30-60 min based on ISWT speeds. The plan to increase PA was based on gradually raising the number of daily steps, inspired by the "theory of establishment of goals or objectives". Each week, participants aimed to increase steps by 10-20% in relation to previous week. Patients used pedometers to control walking speed and number of daily steps. Noted gait and steps in activity	21 (17 analysed)	Community-based	5 sessions/ week	8 weeks	**General PA recommend-ations:**<br><br>Received informative sessions about the benefits of exercise. Given same pedometer as subjects in the experimental group, but no specific instructions, only general recommend-dations to walk more every day.	19 (16 analysed)	Not reported	Not reported	8 weeks

	diary, checked weekly through phone calls.									
Vasilopoulou (2017)	**Centre-based PR / Home-based maintenance group:**<br><br>*Initial 8 weeks centre-based PR:*<br><br>Consisting of interval cycling exercise and resistance training for upper and lower limbs. Patients were provided with dietary advice, instructions on breathing control and self-management education sessions.<br><br>*Home-based PR maintenance:*<br><br>Comprised of a) individualized action plan; b) educational session on self-management; c) physical exercise sessions to remote monitoring; d) access to the call centre; e)	47	Centre- and home-based	Initial centre-based PR (3 sessions/ week)<br><br>Home-based maintenance (144 sessions over 12 months)	*Initial PR:* 8 weeks<br><br>*Maintenance PR:* 12 months	**Centre-based PR / centre-based maintenance group:**<br><br>Initial 8 weeks centre-based PR same as in experimental group.<br><br>*Centre-based maintenance PR:*<br><br>Hospital-based maintenance program included aerobic and resistance exercise regimes (as in initial PR program). Participants also provided with dietary advice, instructions on breathing control and self-management techniques<br><br>**Usual care group**	50<br><br>50	Centre-based	Initial centre-based PR (3 sessions/ week).<br><br>Centre-based maintenance (twice weekly)	Initial PR: 8 weeks<br><br>Maintenance PR: 12 months

	psychological support; f) dietary advice; g) breathing retraining techniques; h) professional scheduled weekly contacts with a physiotherapist, an exercise scientist, a dietician and a physician via telephone or Skype video conference									
Wallaet (2020)	**PR:** Included individual and group-based strengthening exercises, upper/lower limb training, and super-vised endurance training. In addition, patients received training in resumption of daily life physical activity, therapeutic patient education, psychosocial support, and motivational communication.	20 (18 completed)	Centre-based	At least 3 sessions/ week	2 months	**PA counselling:** Received oral counselling to increase their physical activity at home	18 (13 completed)	Unclear	Unclear	2 months

Supplementary material E – Network plot for a) all included articles (n=48), and those reporting changes in b) daily step count, c) time spent in moderate-to-vigorous physical activity, and d) sedentary time

a)

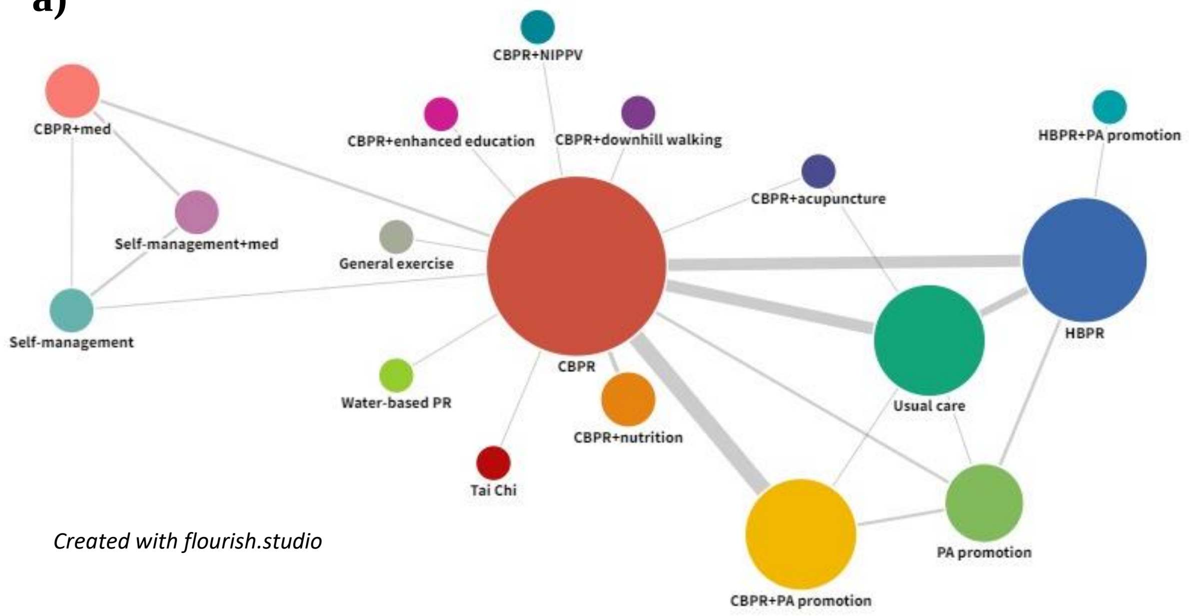

b)

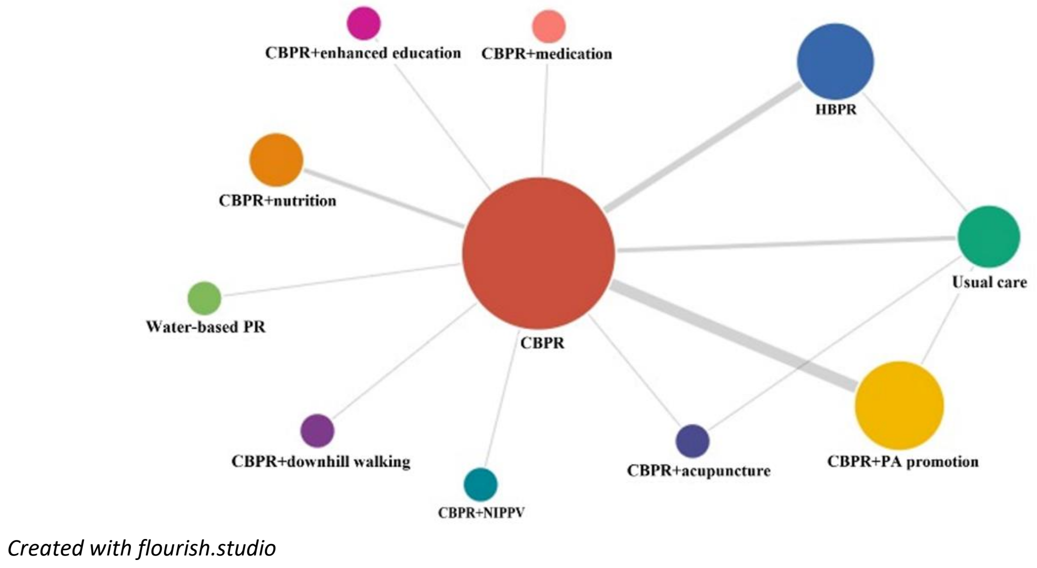

c)

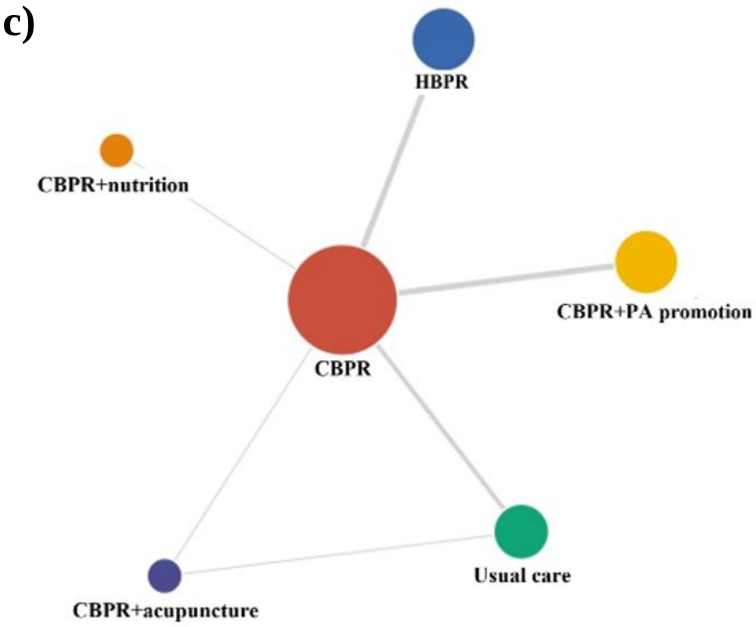

Created with flourish.studio

d)

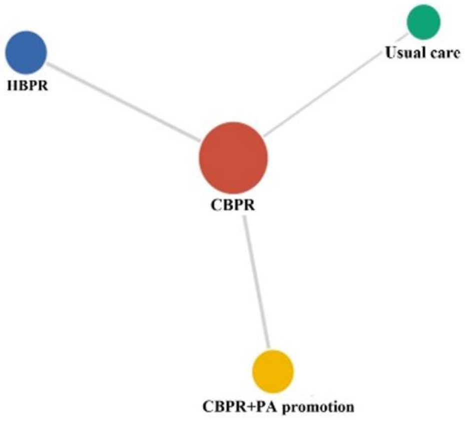

Created with flourish.studio

Note: The circle size within the network plots represents the number of studies that examined an intervention, and the line thickness between interventions represents the number of studies that compared these interventions. *Abbreviations:* CBPR, centre-based pulmonary rehabilitation; HBPR, home-based pulmonary rehabilitation; NIPPV, non-invasive positive pressure ventilation; med, medication; PA, physical activity.

Supplementary material F – Radial plot showing the number of descriptive outcomes reported for physical activity (PA), sedentary behaviour (SB) and sleep quality, connected to the measurement tools used to generate them

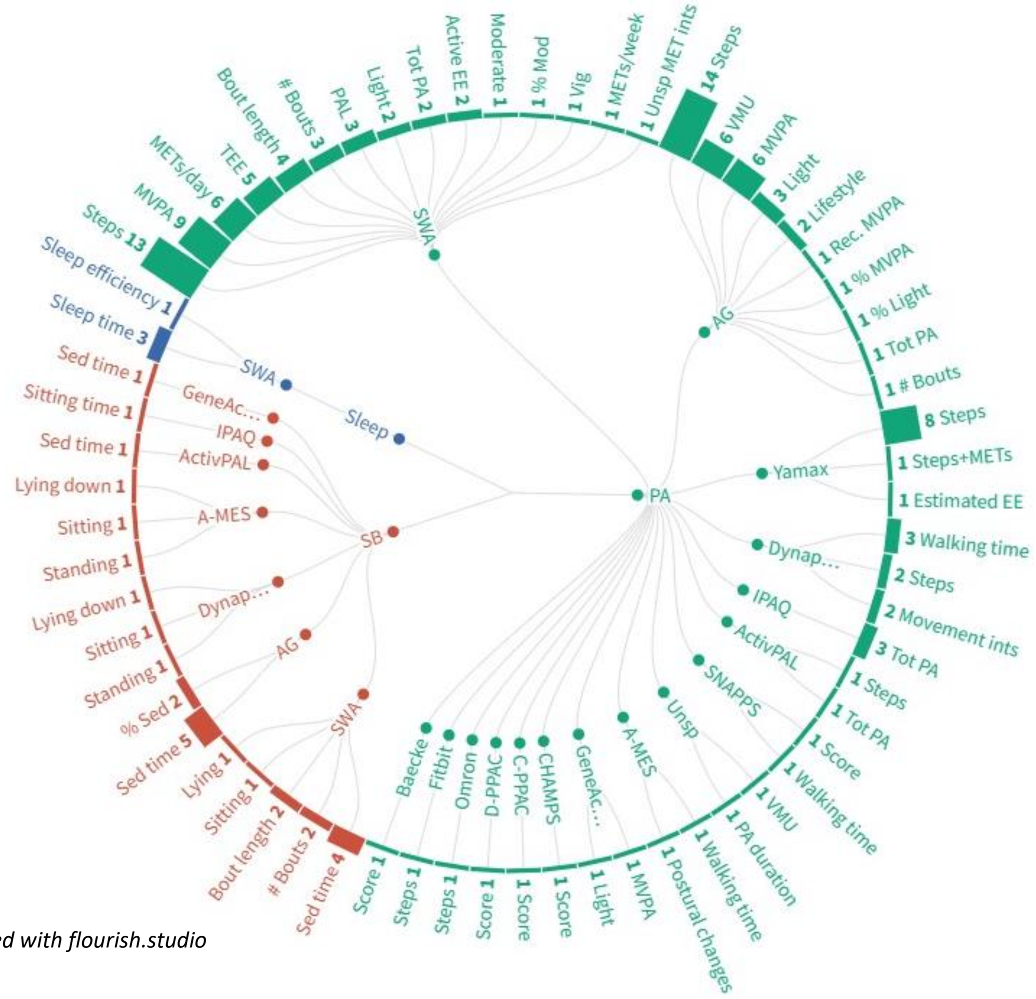

Abbreviations: ActPAL, ActivPAL; AG, ActiGraph; A-MES, Activity Monitoring and Evaluation System; CHAMPS, Community Healthy Activities Model Programme for Seniors; C-PPAC, Clinical PROactive Physical Activity in COPD; Dynap, Dynaport; GeneAc, GeneActiv; IPAQ, International Physical Activity Questionnaire; PA, Physical Activity; SB, Sedentary Behaviour; SNAPPS, Smoking, Nutrition, Alcohol consumption, Physical activity, Psychological well-being, and Symptom management; SWA, SenseWear Armband; Unsp, Unspecified.

Supplementary material G – Primary vs secondary outcomes

Author	Primary outcome(s)	PA/SB/Sleep	Pre-specified power calculation for PA/SB/Sleep
Aldhahir (2021)	ISWT	Secondary	
Altenburg (2015)	Steps/day	Primary	Yes – steps/day
Armstrong (2021)	C-PPAC	Primary	Yes – C-PPAC
Bentley (2020)	Unclear	Secondary	
Breyer (2010)	Daily PA activities	Primary	No
Burge (2021)	Δ6MWD (from Holland)	Secondary	
Burtin (2015)	Walking time and MVPA	Primary	Yes – Walking time
Cameron Tucker (2016)	6MWD	Secondary	
Camillo (2020)	Δ6MWD	Secondary	
Cerdan de las Heras (2021a)	Δ6MWD	Secondary	
Cerdan de las Heras (2021b)	Δ6MWD	Secondary	
Cerdan de las Heras (2022)	Δ6MWD	Secondary	
Cerdeno de Jesus (2022)	ΔVO2 peak	Secondary	
Chaplin (2022)	ISWT (main paper)	Secondary	
Cox (2022)	ΔCRQ-D	Secondary	
Cruz (2016)	MVPA	Primary	Yes - MVPA
Curtis (2016)	Δ peak power	Secondary	
De Blok (2006)	Steps/day	Primary	Unclear
Deering (2011)	Systemic inflammation	Secondary	
Duiverman (2008)	CRQ	Secondary	
Effing (2011)	ISWT	Secondary	
Felcar (2018)	Physical activity in daily life (PADL)	Primary	Unclear
Gaunaurd (2014)	Unclear	Unclear	
Geidl (2021)	Steps/day (between experimental and control)	Primary	Yes – steps/day
Hansen (2020)	Δ6MWD	Secondary	
Holland (2017)	Δ6MWD	Secondary	
Horton (2021)	CRQ-SR (main trial)	Secondary	

Jarosch (2020)	Δ6MWD	Secondary	
Jose (2021)	ISWT	Secondary	
Kawagoshi (2015)	Unclear	Unclear	No
Kesten (2008)	Unclear	Secondary	
Lahham (2020)	Δ6MWD	Secondary	
Louvaris (2016)	Not reported	Unclear	
Nolan (2017)	ΔMVPA	Primary	Yes - MVPA
O'Neill (2018)	Not stated	Unclear	No (yes in discussion for future fully powered trial)
Park (2020)	Self-care behaviour	Secondary	
Pavitt (2020)	ΔISWT	Secondary	
Perez Bogerd (2018)	6MWD	Secondary	
Polgar (2021)	ΔMVPA (from Nolan)	Primary	
Polkey (2018)	ΔSGRQ	Secondary	
Rausch Osthoff (2021)	Steps/day	Primary	No
Selzler (2021)	Steps/day	Primary	Yes – steps/day
Sewell (2005)	Activity monitor counts	Primary	Yes – counts
Troosters (2018)	ESWT	Secondary	
Van de Bool (2017)	Quad muscle strength	Secondary	
Varas (2018)	Unclear	Unclear	
Vasiloupolou (2017)	Rate of exacerbations	Secondary	
Wallaert (2020)	Time spent in activities requiring >2.5 METs	Primary	Yes – time spent in activities requiring >2.5 METs

Supplementary material H – All comparisons between interventions for changes in a) daily step count, b) time spent in MVPA, and c) sedentary time.

a) Changes in daily step count

	CBPR+ medication	CBPR+ acupuncture	Usual care	CBPR+ enhanced education	CBPR	CBPR+ downhill walking	CBPR water-based	HBPR	CBPR+ nutrition	CBPR+PA promotion	CBPR+ NIPPV
**CBPR+ medication**		.	.	.	-943.00 [-2491.99; 605.99]	.	.	.	.	.	.
**CBPR+ acupuncture**	-148.83 [-2090.31; 1792.64]		-365.00 [-1582.89; 852.89]	.	-451.00 [-1759.61; 857.61]	.	.	.	.	.	.
**Usual care**	-263.13 [-1950.05; 1423.79]	-114.30 [-1254.67; 1026.08]		.	-756.42 [-1479.62; -33.22]	.	.	-409.00 [-3161.21; 2343.21]	.	-1115.00 [-2460.62; 230.62]	.
**CBPR+ enhanced education**	-529.00 [-2604.34; 1546.34]	-380.17 [-2190.58; 1430.25]	-265.87 [-1800.14; 1268.40]		-414.00 [-1795.18; 967.18]	.	.	.	.	.	.
**CBPR**	-943.00 [-2491.99; 605.99]	-794.17 [-1964.61; 376.28]	-679.87 [-1347.94; -11.80]	-414.00 [-1795.18; 967.18]		-176.00 [-1779.34; 1427.34]	-676.00 [-2357.18; 1005.18]	-625.67 [-1318.37; 67.04]	-704.45 [-1414.52; 5.62]	-728.66 [-1220.46; -236.85]	-1107.00 [-2648.77; 434.77]
**CBPR+ downhill walking**	-1119.00 [-3348.37; 1110.37]	-970.17 [-2955.27; 1014.94]	-855.87 [-2592.83; 881.09]	-590.00 [-2706.22; 1526.22]	-176.00 [-1779.34; 1427.34]		.	.	.	.	.
**CBPR water-based**	-1619.00 [-3904.99; 666.99]	-1470.17 [-3518.66; 578.33]	-1355.87 [-3164.93; 453.19]	-1090.00 [-3265.79; 1085.79]	-676.00 [-2357.18; 1005.18]	-500.00 [-2823.16; 1823.16]		.	.	.	.
**HBPR**	-1515.26 [-3204.11; 173.59]	-1366.42 [-2705.10; -27.75]	-1252.13 [-2171.90; -332.36]	-986.26 [-2522.65; 550.14]	-572.26 [-1245.19; 100.67]	-396.26 [-2135.09; 1342.58]	103.74 [-1707.12; 1914.60]		.	.	.
**CBPR+ nutrition**	-1647.45	-1498.62 [-2867.61;	-1384.32 [-2359.27;	-1118.45	-704.45 [-1414.52;	-528.45	-28.45	-132.20		.	.

	[-3351.44; 56.54]	-129.63]	-409.38]	[-2671.47; 434.57]	5.62]	[-2282.00; 1225.09]	[-1853.44; 1796.54]	[-1110.48; 846.08]			
**CBPR+PA promotion**	-1639.30 [-3260.80; -17.79]	-1490.46 [-2735.34; -245.59]	-1376.17 [-2143.92; -608.41]	-1110.30 [-2572.34; 351.75]	-696.30 [-1175.78; -216.81]	-520.30 [-2193.80; 1153.21]	-20.30 [-1768.52; 1727.93]	-124.04 [-947.19; 699.11]	8.16 [-848.64; 864.95]		.
**CBPR+ NIPPV**	-2050.00 [-4235.51; 135.51]	-1901.17 [-3836.88; 34.55]	-1786.87 [-3467.16; -106.58]	-1521.00 [-3590.96; 548.96]	-1107.00 [-2648.77; 434.77]	-931.00 [-3155.36; 1293.36]	-431.00 [-2712.11; 1850.11]	-534.74 [-2216.97; 1147.49]	-402.55 [-2099.98; 1294.88]	-410.70 [-2025.31; 1203.91]	

b) Changes in time spent in MVPA

	**CBPR+ acupuncture**	**Usual care**	**CBPR**	**HBPR**	**CBPR+PA promotion**	**CBPR+ nutrition**
**CBPR+ acupuncture**		-37.30 [-78.15; 3.55]	-31.30 [-74.63; 12.03]	.	.	.
**Usual care**	-35.42 [-76.19; 5.35]		-6.49 [-11.57; -1.41]	.	.	.
**CBPR**	-41.91 [-82.90; -0.92]	-6.49 [-11.57; -1.41]		-2.61 [-14.14; 8.91]	-4.59 [-9.43; 0.25]	-14.40 [-34.84; 6.04]
**HBPR**	-44.52 [-87.10; -1.94]	-9.10 [-21.69; 3.49]	-2.61 [-14.14; 8.91]		.	.
**CBPR+PA promotion**	-46.50 [-87.77; -5.22]	-11.08 [-18.10; -4.06]	-4.59 [-9.43; 0.25]	-1.98 [-14.48; 10.52]		.
**CBPR+ nutrition**	-56.31 [-102.11; -10.50]	-20.89 [-41.95; 0.17]	-14.40 [-34.84; 6.04]	-11.79 [-35.25; 11.68]	-9.81 [-30.81; 11.20]	

c) Changes in sedentary time

	**CBPR+ PA promotion**	**HBPR**	**CBPR**	**Usual care**
**CBPR+ PA promotion**		.	-21.16 [-50.76; 8.43]	.
**HBPR**	-2.64 [-54.14; 48.85]		-18.52 [-60.65; 23.62]	.
**CBPR**	-21.16 [-50.76; 8.43]	-18.52 [-60.65; 23.62]		-48.30 [-79.77; -16.83]
**Usual care**	-69.46 [-112.66; -26.26]	-66.82 [-119.41; -14.22]	-48.30 [-79.77; -16.83]	

Note: Values above blacked out diagonal line are estimates from pairwise meta-analyses (direct), values below the diagonal line are estimates from network meta-analyses (both direct and indirect). Comparisons that are shaded show statistically significant differences between interventions.

Supplementary material I – Sensitivity analyses: correlation coefficient of 0.5 when imputing SDs for a) daily step count, b) time spent in moderate-to-vigorous physical activity, and c) sedentary time

a)

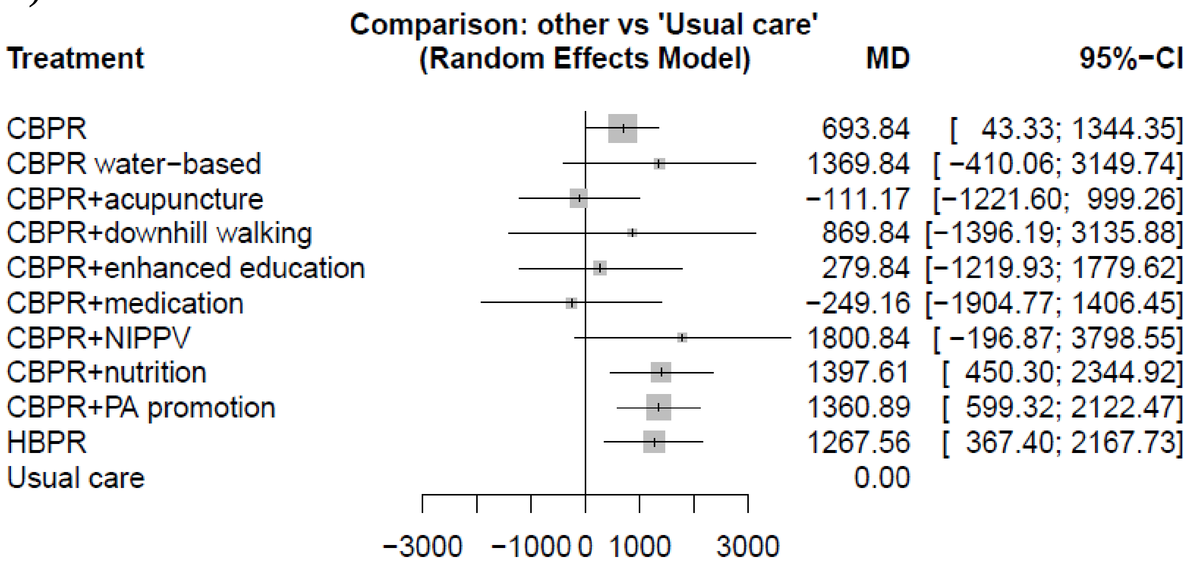

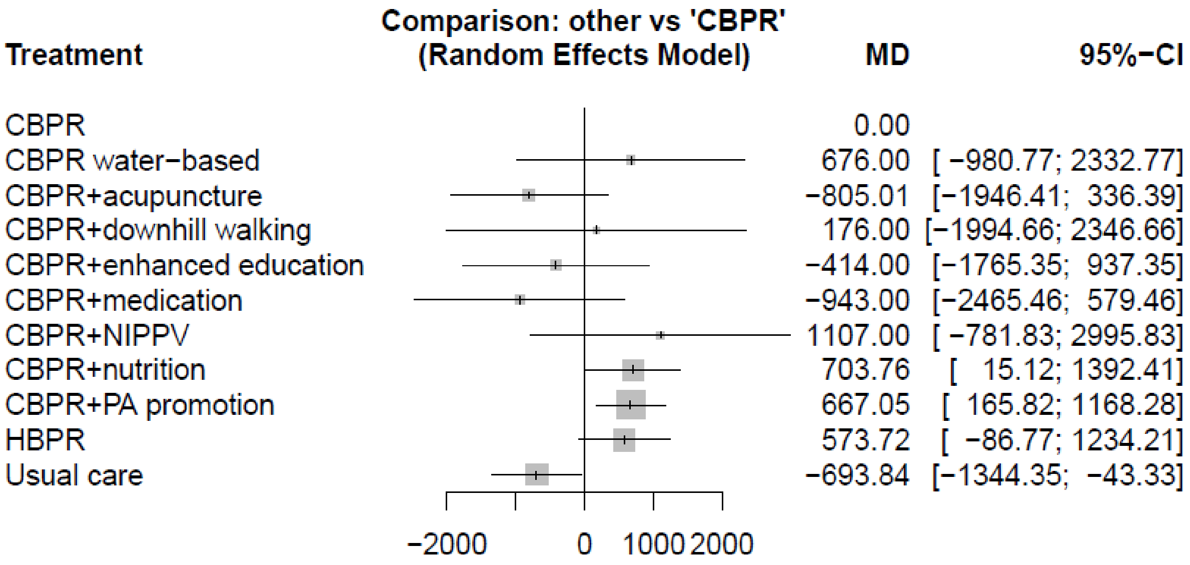

b)

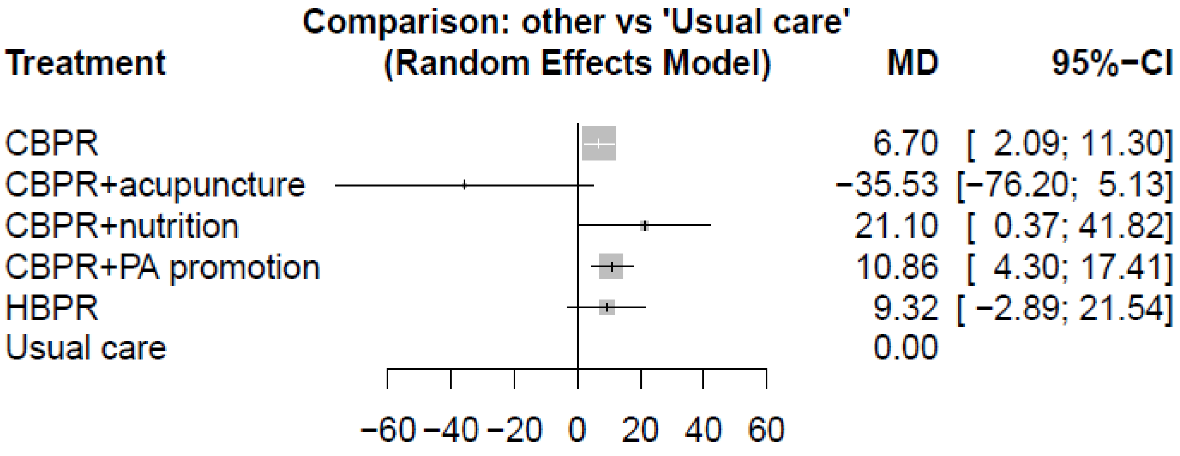
Comparison: other vs 'Usual care'
(Random Effects Model)
Treatment
MD
95%−CI
CBPR 6.70 [2.09; 11.30]
CBPR+acupuncture −35.53 [−76.20; 5.13]
CBPR+nutrition 21.10 [0.37; 41.82]
CBPR+PA promotion 10.86 [4.30; 17.41]
HBPR 9.32 [−2.89; 21.54]
Usual care 0.00
−60 −40 −20 0 20 40 60

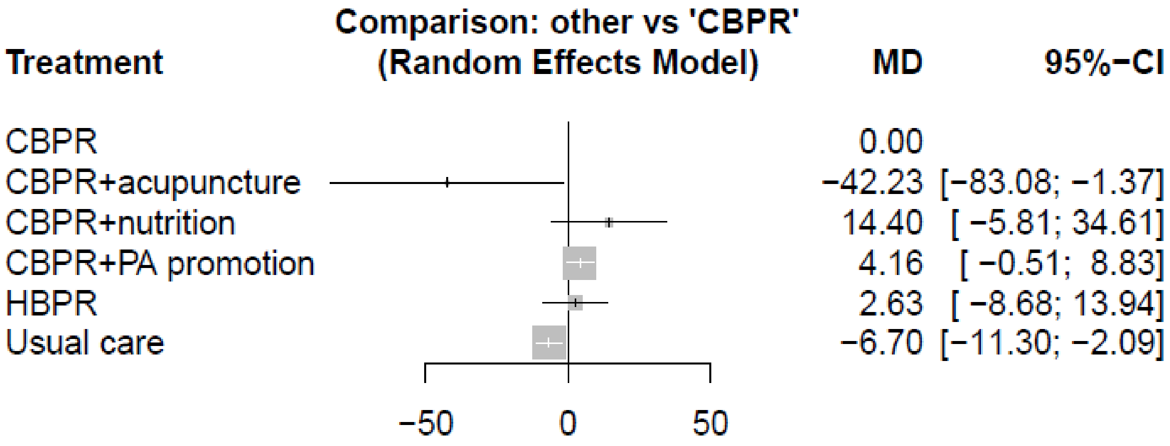
Comparison: other vs 'CBPR'
(Random Effects Model)
Treatment
MD
95%−CI
CBPR 0.00
CBPR+acupuncture −42.23 [−83.08; −1.37]
CBPR+nutrition 14.40 [−5.81; 34.61]
CBPR+PA promotion 4.16 [−0.51; 8.83]
HBPR 2.63 [−8.68; 13.94]
Usual care −6.70 [−11.30; −2.09]
−50 0 50

c)

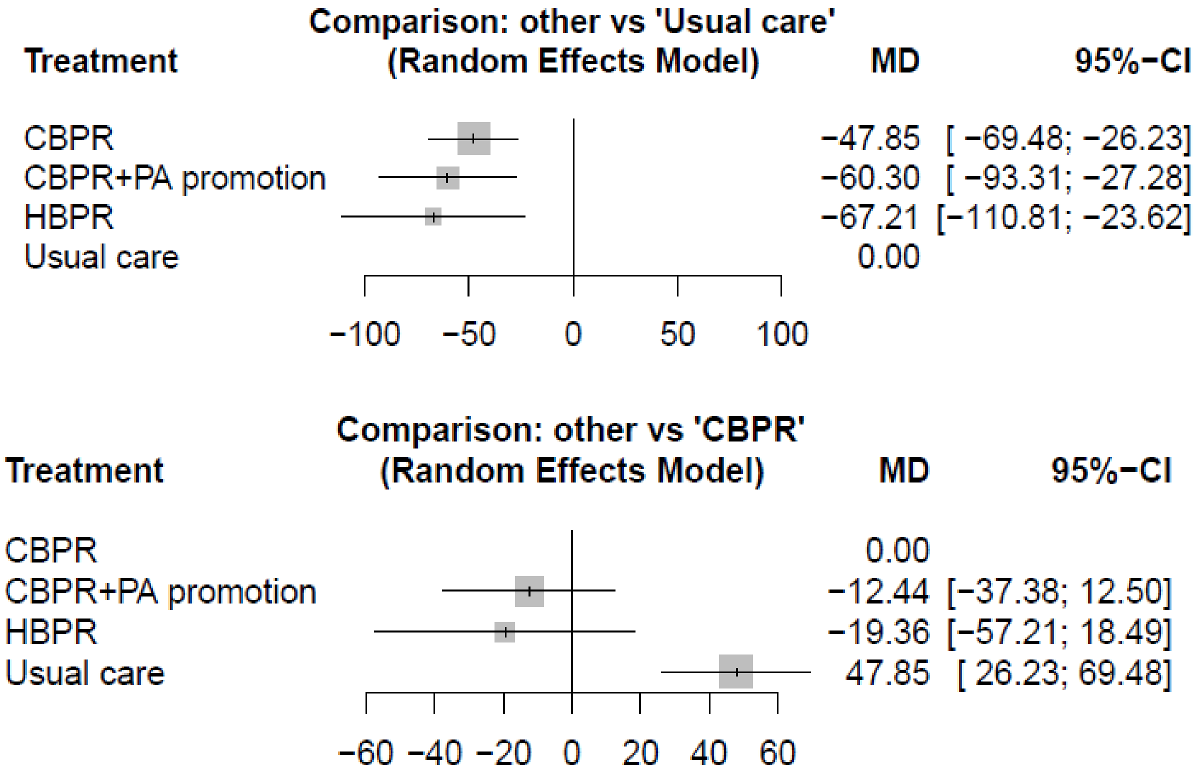
Comparison: other vs 'Usual care'
(Random Effects Model)
Treatment
MD
95%−CI
CBPR
CBPR+PA promotion
HBPR
Usual care
−47.85 [−69.48; −26.23]
−60.30 [−93.31; −27.28]
−67.21 [−110.81; −23.62]
0.00
−100 −50 0 50 100
Comparison: other vs 'CBPR'
(Random Effects Model)
Treatment
MD
95%−CI
CBPR
CBPR+PA promotion
HBPR
Usual care
0.00
−12.44 [−37.38; 12.50]
−19.36 [−57.21; 18.49]
47.85 [26.23; 69.48]
−60 −40 −20 0 20 40 60

Supplementary material J – Sensitivity analyses: Primary vs secondary outcomes for a) daily step count, and b) time spent in moderate-to-vigorous physical activity

a)

Primary outcome (k=5)

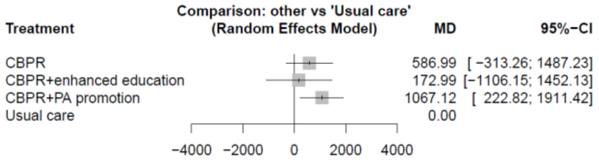

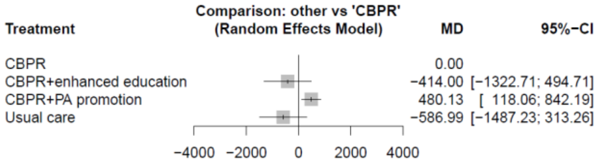

Secondary outcome (k=19)

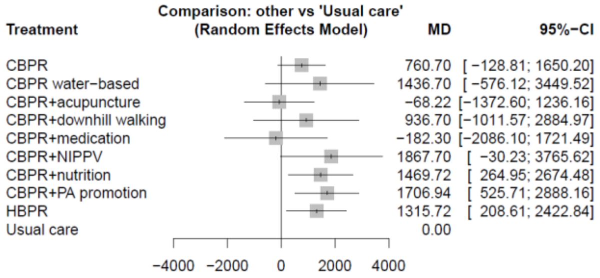

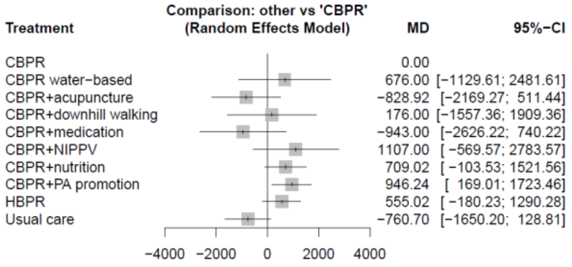

b)

Primary outcome (k=2)

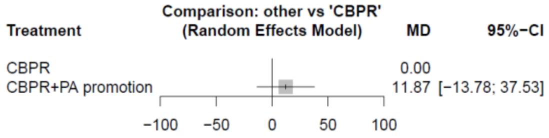

Secondary outcome (k=10)

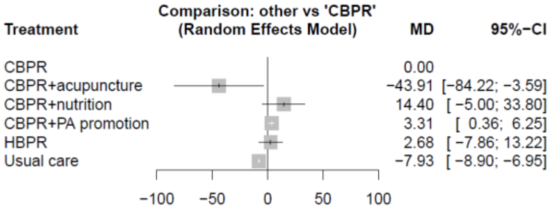

Supplementary material K - Risk of Bias 2 results for studies using device-based measures of movement behaviours

Study ID	D1	D2	D3	D4	D5	Overall
Aldhahir 2021	+	+	!	+	!	!
Altenburg 2015	+	+	-	+	!	-
Armstrong 2021	!	+	-	+	!	-
Breyer 2010	+	+	+	-	!	-
Burtin 2015	+	+	-	+	!	-
Burtin 2015	+	+	-	+	!	-
Camillo 2020	+	+	-	+	!	-
Cerdan-de-las-Heras 2021a	+	+	-	-	!	-
Cerdan-de-las-Heras 2021b	+	+	-	+	!	-
Cerdan-de-las-Heras 2022	+	+	-	+	!	-
Cedeno de Jesus 2022	+	+	+	+	+	+
Chaplin 2022	+	+	-	+	!	-
Cox 2022	+	!	+	+	+	!
Cruz 2016	+	+	!	+	!	!
Curtis 2016	+	+	-	+	!	-
de Blok 2006	!	+	-	+	!	-
Deering 2011	+	+	-	+	!	-
Duiverman 2008	+	+	!	!	!	!
Effing 2011	+	+	!	+	!	!
Felcar 2018	+	+	!	+	!	!
Geidl 2021	+	+	+	+	!	!
Hansen 2020	+	+	-	+	+	-
Holland 2017	+	+	-	+	+	-
Horton 2021	+	+	-	+	!	-
Jarosch 2020	+	+	+	+	!	!
Jose 2021	+	+	-	+	!	-
Kawagoshi 2015	-	+	-	+	!	-
Lahham 2020	+	+	!	+	!	!
Louavaris 2016	+	+	-	+	!	-
Nolan 2017	+	+	-	+	!	-
O'Neill 2018	+	!	-	+	-	-
O'Neill 2018	+	!	-	+	-	-
Park 2020	+	+	+	+	!	!
Pavitt 2020	+	+	!	+	!	!
Perez-Bogerd 2018	+	+	-	+	!	-
Polkey 2018	!	!	-	+	!	-
Rausch Osthoff 2021	+	-	!	+	+	-
Selzler 2021	+	!	!	+	!	!
Sewell 2005	+	+	-	+	!	-
Troosters 2018	+	+	-	+	+	-

+ Low risk
! Some concerns
- High risk

D1	Randomisation process
D2	Deviations from the intended interventions
D3	Missing outcome data
D4	Measurement of the outcome
D5	Selection of the reported result

van de Bool 2017	+	+	!	+	!	!
Varas 2018	+	+	-	+	!	-
Vasilopoulou 2017	+	+	+	+	!	!
Wallaert 2020	+	+	-	+	!	-

Supplementary material L - Risk of Bias 2 results for studies using questionnaire-based measures of movement behaviours

Study ID	D1	D2	D3	D4	D5	Overall
Armstrong 2021	!	+	-	-	!	-
Bentley 2020	+	+	-	-	!	-
Cameron-Tucker 2016	+	+	+	+	!	!
Gaunaurd 2014	+	+	!	!	!	!
Kesten 2008	!	+	-	+	!	-
O'Neill 2018	+	!	-	!	-	-
Rausch Osthoff 2021	+	-	!	+	+	-
Troosters 2018	+	+	-	!	+	-
Varas 2018	+	+	-	+	!	-

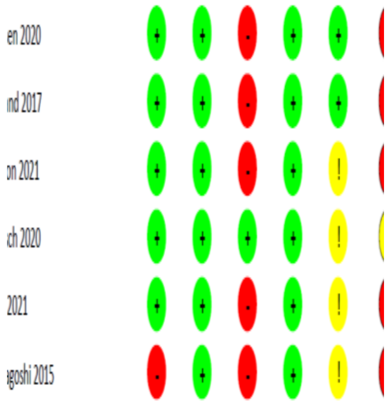

Supplementary material M - GRADE results for a) daily step count, b) time spent in MVPA, and c) sedentary time

a)

GRADE criteria	Rating	Footnotes	Quality of the evidence
**Outcome:** Daily step count			
**Study design**	High	All studies (n=24) were randomised trials of which 20 were RCTs.	
**Risk of bias**	Serious (-1)	The majority of these studies were deemed to have high overall risk of bias using the RoB2 tool (15/24). No difference in findings when sensitivity analysis performed using only low risk studies for D4: measurement of the outcome	
**Inconsistency**	No	11 different interventions were included within the NMA; however, this was the justification for performing a NMA over a traditional MA.	
**Indirectness**	No	All studies included COPD. Differences in interventions and comparisons were the justification of performing an NMA. All studies (100%) appropriately measured this outcome (RoB2 4.1).	Low
**Imprecision**	Serious (-1)	Daily step count was the primary outcome in only 5/24 studies. Wide CIs in NMA effect estimates.	
**Publication bias**	Undetected	Unlikely to contribute to publication bias.	
**Other**			

b)

GRADE criteria	Rating	Footnotes	Quality of the evidence
**Outcome:** Time spent in moderate-to-vigorous physical activity (MVPA)			
**Study design**	High	All studies (n=12) were randomised trials of which 10 were RCTs.	
**Risk of bias**	Serious (-1)	The majority of these studies were deemed to have high overall risk of bias using the RoB2 tool (7/12). All studies within NMA low risk for D4: measurement of the outcome, so unable to perform sensitivity analysis.	
**Inconsistency**	No	6 different interventions were included within the NMA; however, this was the justification for performing a NMA over a traditional MA.	Low
**Indirectness**	No	All studies included COPD. Differences in interventions and comparisons were the justification of performing an NMA. All studies (100%) appropriately measured this outcome (RoB2 4.1).	
**Imprecision**	Serious (-1)	MVPA was the primary outcome in only 2/12 studies. Wide CIs in NMA effect estimates.	
**Publication bias**	Undetected	Unlikely to contribute to publication bias.	
**Other**			

c)

GRADE criteria	Rating	Footnotes	Quality of the evidence
**Outcome:** Sedentary time			
**Study design**	High	All studies (n=7) were RCTs.	Low
**Risk of bias**	Serious (-1)	Overall RoB proportion of sedentary time studies within NMA is high. All studies within NMA low risk for D4: measurement of the outcome, so unable to perform sensitivity analysis.	
**Inconsistency**	No	4 different interventions were included within the NMA; however, this was the justification for performing a NMA over a traditional MA.	
**Indirectness**	No	All studies included COPD. Differences in interventions and comparisons were the justification of performing an NMA. All studies (100%) appropriately measured this outcome (RoB2 4.1).	
**Imprecision**	Serious (-1)	Sedentary time was not reported as a primary outcome in any study. Wide CIs in NMA effect estimates.	
**Publication bias**	Undetected	Unlikely to contribute to publication bias.	
**Other**			

Supplementary material N – TIDieR results for individual studies (1, provided; 0, not provided)

Studies	Item 1	Item 2	Item 3	Item 4	Item 5	Item 6	Item 7	Item 8	Item 9	Item 10	Item 11	Item 12	Total
Aldhahir (2021)	1	1	1	1	1	0	1	1	0	0	1	1	9
Altenburg (2015)	1	1	1	0	1	1	1	1	0	0	0	0	7
Armstrong (2021)	1	1	1	1	1	1	1	1	1	1	1	1	12
Bentley (2020)	1	1	1	0	0	1	1	1	0	0	1	1	8
Breyer (2010)	1	1	1	1	1	1	0	1	1	0	1	1	10
Burtin (2015)	1	1	1	1	0	1	1	1	1	0	0	0	8
Cameron-Tucker (2016)	1	1	1	1	1	1	1	1	1	0	1	1	11
Camillo (2020)	1	1	1	1	0	1	1	1	0	0	1	1	9
Cedeño de Jesús (2022)	1	1	1	1	1	1	1	1	0	0	1	1	10
Cerdan-de-las-Heras (2021a)	1	1	1	1	1	1	1	1	0	0	1	1	10
Cerdan-de-las-Heras (2021b)	1	1	1	1	1	1	1	1	1	0	1	1	11
Cerdan-de-las-Heras (2022)	1	1	1	1	1	1	1	1	1	0	1	1	11
Chaplin (2022)	1	1	1	1	0	1	1	1	0	0	0	0	7
Cox (2022)	1	1	1	1	1	1	1	1	1	0	1	1	11
Cruz (2016)	1	1	1	1	1	1	0	1	1	1	0	0	9
Curtis (2016)	1	1	1	1	1	1	0	1	1	0	0	1	9
de Blok (2006)	1	1	1	0	1	0	0	1	0	0	0	1	6
Deering (2011)	1	1	1	1	0	1	0	1	0	0	0	0	6
Duiverman (2008)	1	1	1	1	0	1	0	1	0	0	0	1	7
Effing (2011)	1	1	1	1	1	1	1	1	1	0	0	1	10
Felcar (2018)	1	1	1	1	1	1	1	1	1	0	0	1	10
Gaunaurd (2014)	1	1	1	1	0	1	0	1	0	0	0	0	6
Geidl (2021)	1	1	1	1	1	1	1	1	0	0	0	0	8
Hansen (2020)	1	1	1	1	1	1	1	1	1	0	1	1	11
Holland (2017) / Burge (2021)	1	1	1	1	1	1	1	1	1	0	1	1	11
Horton (2021)	1	1	1	1	1	1	1	1	0	0	0	0	8
Jarosch (2020)	1	1	1	1	0	1	1	1	1	0	0	0	8
Jose (2021)	1	1	1	1	1	1	1	1	1	0	1	0	10
Kawagoshi (2015)	1	1	1	1	1	1	1	1	0	0	1	0	9
Lahham (2020)	1	1	1	1	1	1	1	1	1	0	1	1	11
Kesten (2008)	1	1	1	1	0	1	0	1	0	0	0	0	6

Louvaris (2016)	1	1	1	1	0	0	0	1	1	0	0	0	6
Nolan (2017) / Polgar (2021)	1	1	1	1	1	1	1	1	1	0	0	0	9
O'Neill (2018)	1	1	1	1	1	1	1	1	1	1	1	1	12
Park (2020)	1	1	1	1	1	1	1	1	1	1	1	1	12
Pavitt (2020)	1	1	1	0	0	1	1	1	0	0	1	1	8
Perez-Bogerd (2018)	1	1	1	1	1	1	1	1	1	0	0	1	10
Polkey (2018)	1	1	1	1	1	1	1	1	1	0	0	1	10
Rausch Osthoff (2021)	1	1	1	1	1	1	1	1	1	1	1	1	12
Selzler (2021)	1	1	1	1	1	1	1	1	1	0	1	1	11
Sewell (2005)	1	1	1	1	0	1	1	1	1	0	0	0	8
Troosters (2018)	1	1	1	1	0	1	1	1	1	0	1	1	10
van de Bool (2017)	1	1	1	1	1	1	1	1	1	0	1	1	11
Varas (2018)	1	1	1	1	0	1	1	1	1	0	1	0	9
Vasilopoulou (2017)	1	1	1	1	1	1	1	1	1	0	1	1	11
Wallaert (2020)	1	1	0	0	0	1	1	1	0	0	0	1	6

Supplementary material O – Quality and reporting details of device deployment for studies using device-based measures of movement behaviours

Author (Year)	Model of device	Body Location worn	Real-time feedback provided to participants	Period of wear	Valid wear time requirement	Fixed or minimum wear time	Number of valid days/nights required	Fixed or minimum valid days	Non-wear detection/ description of identifying missing data or device taken off	Average wear time	Waking wear time calculable	Outcome variables	Definitions of outcomes
Accelerometer													
Armstrong (2021)	ActiGraph wGT3X	Not reported	N/A	7 conse-cutive days	8 hours	Minimum	4 weekdays	Minimum	Not reported	Not reported	Yes	C-PPAC total score<br><br>C-PPAC amount score<br><br>Step count (steps/day)<br><br>Movement intensity<br><br>Sedentary time (min)<br><br>Light time (min)<br><br>MVPA time (min)	Movement intensity: VMU<br><br>Sedentary time: Not stated<br><br>Light time: Not stated<br><br>MVPA time: Not stated
Breyer (2010)	DynaPort Activity Monitor	Waist and upper leg	N/A	3 conse-cutive week days	12 hours	Fixed	Not reported	Not reported	Not reported	Not reported	Yes	Movement intensity<br><br>Walking time (min/day)<br><br>Standing time (min/day)<br><br>Sitting time (min/day)	Movement intensity: m/s^2

												Lying time (min/day)	
Burge (2021)	SenseWear armband	Left upper arm	N/A	1 week	10 hours	Minimum	4 days (inclusive of at least 1 weekend day)	Minimum	Not reported	Baseline: Centre = 1420 [1401, 1432], home = 1426 [1411, 1430]<br><br>End-rehab: Centre = 1417 [1377, 1427], home = 1427 [1418, 1434]<br><br>Follow-up: 1405 [1315, 1426], home = 1418 [1361, 1430]	Yes	Sedentary time<br><br>Light intensity PA time<br><br>MVPA time<br><br>Sleep time	Sedentary time: awake, ≤1.5 METs<br><br>Light intensity PA time: >1.5 to <3METs<br><br>MVPA time: ≥3METs
Burtin (2015)	Minimod	Lower back (height of second lumbar vertebra, nearby body's centre of mass)	N/A	7 conse-cutive days	8 hours	Minimum	Not reported	Not reported	Not reported	Not reported	No	Daily walking time<br><br>Step count (steps/day)	
	SenseWear	Right upper arm	N/A	7 conse-cutive days	8 hours	Minimum	Not reported	Not reported	Not reported	Not reported	No	Time spent in at least moderate intense activity<br><br>Time spent in at least mild intense activity	At least moderate intense activity time: > 3.6 METs<br><br>At least mild intense activity time: > 2.0 METs
Camillo (2020)	Actigraph wGT3X	Not reported	N/A	1 week	8 hours*	Minimum *	4 days*	Minimum *	Not reported	Not reported	No	Step count (steps/day)	
Cedeño de Jesús (2022)	SenseWear mini armband	Right triceps	N/A	5 days	Not reported	Not reported	Not reported	Not reported	Not reported	Yes (hours)<br><br>Baseline: HBPR = 23.32 ± 0.28,	No	Step count (steps/day)<br><br>METs	

										control = 22.34 ± 2.58<br><br>Post: HBPR = 22.85 ± 1.71, control = 22.64 ± 1.85		Lying time<br><br>Sleep time	
Cerdan-de-las-Heras (2021a)	Actigraph wGT3X-BT	Not reported	N/A	7 days	Not reported	Not reported	Not reported	Not reported	Not reported	Not reported	No	Step count (steps/day)<br><br>Total vector magnitude counts per minute (VMC/min)	
Cerdan-de-las-Heras (2021b)	Actigraph wGT3X-BT	Not reported	N/A	7 days	Not reported	Not reported	Not reported	Not reported	Not reported	Not reported	No	Step count (steps/day)<br><br>VMCPM	
Cerdan-de-las-Heras (2022)	Actigraph wGT3X-BT	Not reported	N/A	7 days	Not reported	Not reported	Not reported	Not reported	Not reported	Not reported	No	Step count (steps/day)<br><br>VMCPM	
Chaplin (2022)	SenseWear	Not reported	N/A	7 days	8 hours	Minimum	4 days	Minimum	Not reported	Not reported	No	Step count (steps/day)<br><br>Daily METs<br><br>Percentage of moderate activity<br><br>Mean bout length of moderate PA<br><br>Number of 2-min bouts of moderate PA<br><br>Number of 5-min bouts of moderate PA<br><br>Number of 10-min bouts of moderate PA	Light PA: >1.5 METs<br><br>Moderate PA: 3-6 METs<br><br>Vigorous: >6 METs

												Number of 20-min bouts of moderate PA<br><br>Total MVPA	
Cox (2022)	GeneActiv	Wrist	N/A	7 days	10 hours	Minimum	4 days	Minimum	Yes. "minute-by-minute outputs for all wear time, including average metabolic equivalent (MET) values for each minute of wear and identification of non-wear time and time spent in bed."	Not reported	Yes	Light intensity PA (min/day)<br><br>MVPA (min/day)	Light PA: ≥1.5-2.99 METs<br><br>MVPA: ≥3METs
Cruz (2016)	Actigraph wGT3X+	Not reported	N/A	4 conse-cutive week days	8 hours	Minimum	Not reported	Not reported	Not reported	Not reported	Yes	Sedentary activity (min/day)<br><br>Total PA (min/day)<br><br>Overall MVPA (min/day)<br><br>Recommended MVPA (min/day)<br><br>Step count (steps/day)	Sedentary: 0-99 counts-per-minute (CPM)<br><br>Total PA: 100-∞ CPM<br><br>MVPA: 1952-∞ CPM<br><br>Time spent in MVPA was calculated considering the total time (overall MVPA) and the internationally recommended duration of 30 min of daily MVPA, either continuous or in blocks of 10 min (recommended MVPA)

Curtis (2016)	SenseWear pro armband	Over body of triceps muscle of right arm	N/A	7 days	Not reported	Not reported	5 days (2 weekend)	Fixed	Not reported	Not reported	No	Step count (steps/day)<br><br>Physical activity level (PAL)	PAL calculated using total EE and sleep expenditure as a surrogate for resting EE (PAL = TEE/REE)
Deering (2011)	SenseWear pro armband	Over the right triceps	N/A	7 conse-cutive days	23 hours	Minimum	Not reported	Not reported	Not reported	Not reported	No	Total energy expenditure (kcal/day)<br><br>PA duration >3 METs<br><br>METs (n)<br><br>Step count (steps/day)<br><br>Sleep efficiency (min)<br><br>Sleep time (min/day)	Sleep efficiency = sleep time / lying down time
Geidl (2021)	Actigraph wGT3X-BT	Right hip*	N/A	7 days*	10 hours*	Minimum *	5 days*	Minimum *	Periods of non-wear time logged each day in activity diary*	Not reported	No	Sedentary time (min/day)<br><br>Moderate intensity PA (min/day)<br><br>Step count (steps/day)	Sedentary time: 0 to ≤100 activity counts<br><br>Moderate PA: >1952 to 5724 activity counts<br><br>*
Hansen (2020)	activePAL	Front of thigh	N/A	5 days	Not reported	Not reported	Not reported	Not reported	Not reported	Not reported	No	Sedentary time<br><br>Active time	Sedentary time: time spent sitting/lying; thigh in horizontal position<br><br>Active time: time spent

												Step count (steps/day)	standing/walking; thigh in vertical position
Holland (2017)<br><br>*As in Burge (2021)*	SenseWear armband	Left upper arm	N/A	1 week	10 hours	Minimum	4 days (inclusive of at least 1 weekend day)	Minimum	Not reported	Not reported	No	Sedentary time (min/day)<br><br>Sedentary awake time (min/day)<br><br>Sedentary bouts (n/day)<br><br>Time spent in sedentary bouts (min/day)<br><br>MVPA (min/day)<br><br>MVPA bouts (n/day)<br><br>Time spent in MVPA bouts (min/day)<br><br>Steps/day<br><br>Total energy expenditure<br><br>METs/day	Sedentary time: awake, ≤1.5 METs<br><br>MVPA time: ≥3METs
Horton (2021)	SenseWear armband	Not reported	N/A	5 days (incl-uding 2 week-end days)	12 hours	Minimum	3 days	Minimum	Not reported	Not reported	Yes	Sedentary time<br><br>Light PA<br><br>MVPA<br><br>Vigorous PA<br><br>Energy expenditure >3 METs	Moderate PA: >3 METs

												Time >3 METs in bouts<br><br>Step count (steps/day)	
Jarosch (2020)	SenseWear armband	Not reported	N/A	7 conse-cutive days	23 hours	Minimum	Not reported	Not reported	Not reported	Not reported	No	Step count (steps/day)	
José (2021)	ActiGraph wGT3X-BT	Not reported	N/A	7 conse-cutive days	Not reported	Not reported	Not reported	Not reported	Not reported	Not reported	No	Daily steps (steps/day)	
Kawagoshi (2015)	Activity Monitoring and Evaluation System (A-MES)	Thigh and chest (wearing clothing with 2 pockets)	N/A	Maxi-mum of 7 conse-cutive days	12 hours	Fixed	At least 2 days	Minimum	Not reported	Not reported	No	Time spent walking (min/day)<br><br>Time spent standing (min/day)<br><br>Time spent sitting (min/day)<br><br>Time spent lying down (min/day)<br><br>Frequency of total postural changes (times/day)<br><br>Frequency of getting up (times/day)<br><br>Frequency of standing up (times/day)	Getting up: bodily change from a recumbent position to an upright position<br><br>Standing up: bodily change from a sitting position to an upright position

Lahham (2020)	SenseWear armband	Upper left arm	N/A	7 days	10 hours	Minimum	4 days (inclusive of at least 1 weekend day)	Minimum	Not reported	Not reported	No	Sedentary time (min/day)<br><br>Bouts of sedentary time awake ≥10 mins (n/day)<br><br>Time spent in sedentary bouts (min/day)<br><br>Total energy expenditure<br><br>Average MET (METs/day)<br><br>Steps/day<br><br>MVPA time (min/day)<br><br>Bouts of MVPA ≥10 mins (n/day)<br><br>Time spent in MVPA bouts (min/day)	Sedentary: ≤ 1.5 METs<br><br>Bout = a minimum of 10 continuous minutes of time spent in the specified level of PA<br><br>MVPA: ≥ 3 METs
Louvaris (2016)	Actigraph GT3X	Not reported	N/A	7 conse-cutive days	480 minutes (during waking hours [07:00-20:00])	Minimum	4 days	Minimum	Not reported	Baseline: PR = 765±39, usual care = 755±41<br><br>Post: PR = 759±49, usual care = 749±40	Yes	Sedentarism (%)<br><br>Steps count (steps/day)<br><br>VMU<br><br>Time spent in sedentary activities<br><br>Time spent in light activities	Sedentarism defined as step count <5000 steps/day<br><br>VMU used to quantify daily activity levels (not reported how)

												Time spent in lifestyle activities<br><br>Time spent in MVPA	
Nolan (2017)	SenseWear	Not reported	N/A (did, however, note pedometer measured step count in trial diary; see below)	7 days	22.5 days (is advocated)*	Minimum	">5 days is advocated"*	Minimum	Not reported	Not reported	No	Moderate intensity PA<br><br>Step count (steps/day)	Moderate intensity PA: ≥3 METs (MVPA)
O'Neill (2018)	Actigraph GT3X+	Waist	N/A	7 days	10 hours	Minimum	5 days	Minimum	Not reported	Not reported	No	Step count (steps/day)<br><br>Total MVPA<br><br>MVPA 10+ number of bouts<br><br>MVPA 10+ time	Not reported
Park (2020)	ActiGraph wGT-3X-BT	Waist, right hip	N/A	7 conse-cutive days	10 hours	Minimum	4 days	Minimum	Yes. Non-wear time defined as no counts for 60 min with tolerance up to 2 min of activity between 0 and 100 cpm	Not reported	No	Step count (steps/day)<br><br>Total activity count/wear time<br><br>LPA % time<br><br>MPA % time<br><br>Sedentary activity % time	Time spent for each activity / total wear time
Pavitt (2020)	SenseWear pro	Over bulk of triceps on upper left arm	N/A	7 days	22.5 hours (94%)	Minimum	5 consecutive days (including 2 weekend days). First and last days of	Not reported	Not reported	Not reported	No	Physical activity level (PAL)<br><br>Step count (steps/day)<br><br>Time >3 METs	Moderate activity: >3 METs<br><br>PAL: estimated total daily expenditure divided by estimated whole-

							wearing not included.						night sleeping energy expenditure<br><br>PAL definitions: active ≥ 1.70; sedentary 1.40-1.69; very inactive < 1.40
Perez-Bogerd (2018)	SenseWear armband	Not reported	N/A	7 conse-cutive days	8 hours	Minimum	2 days (weekdays)	Minimum	Not reported	Not reported	No	Step count (steps/day)<br><br>MPA	MPA: >3 METs
Polkey (2018)	ActiGraph (unspecified)	Not reported	N/A	7 days	Not reported	Not reported	Not reported	Not reported	Not reported	Not reported	No	Step count (steps/day)	
Rausch Osthoff	SenseWear armband	Upper right arm*	N/A	7 conse-cutive days*	22.5 hours	Minimum	4 days	Minimum	Not reported	Not reported	Yes	Step count (steps/day)<br><br>Total energy expenditure per day<br><br>MET per day<br><br>PAL per day<br><br>Time with low MET<br><br>Time with medium MET<br><br>Time with high MET<br><br>Time with very high MET<br><br>Average sitting time	PAL calculated using total energy expenditure and sleep expenditure as a surrogate for resting energy expenditure (PAL = TEE/REE)<br><br>MET levels not defined
Selzler (2021)	Fitbit Flex or Fitbit Flex2	Non-dominant wrist	Not reported	1 week	First 10 hours of data recorded	Fixed	5 consecutive days	Not reported	Not reported	Not reported	No	Step count (steps/day)	

Sewell (2005)	Ambulatory activity monitor (Z80-32k)	Waist	N/A	2 conse-cutive days	12 hours (9am-9pm)	Fixed	2 consecutive days	Fixed	Not reported	Not reported	No	Activity monitor counts	For each minute, the intensity of activity was expressed as an arbitrary numerical value ranging from 0 (no activity) to 253 (suprathreshold). The activity level for each patient was expressed as total activity counts for the whole of the 2-day assessment period.
Troosters (2018)	DynaPort MoveMonitor	Not reported	N/A	7 conse-cutive days	Not reported. "during waking hours"	Not reported	Not reported	Not reported	Not reported	Not reported	No	Step count (steps/day)<br><br>D-PPAC total (score)<br><br>Daily walking time (min/day)<br><br>Walking intensity (min/day)	
Van de Bool (2017)	Actigraph GT3X+	Waist	N/A	7 conse-cutive days	Not reported	Not reported	5 days	Minimum	Not reported	Not reported	No	Step count (steps/day)	
Vasilopoulou (2017)	ActiGraph GT3X	Waist above right hip	N/A	Not report-ed	8 hours	Minimum	4 days (including weekends)	Minimum	Not reported	Yes (baseline only)	Yes	Step count (steps/day)<br><br>Sedentary time<br><br>Light time<br><br>Lifestyle time<br><br>Moderate time	

Wallaert (2020)	SenseWear Pro armband	Not reported	N/A	5 consecutive days (2 weekend days)	"instructed to wear the device continuously"	Not reported	Not reported	Not reported	Not reported	Not reported	No	Step count (steps/day)<br><br>Total EE (kcal/day)<br><br>EE > 2.5 METs (kcal/day)<br><br>Time in EE > 2.5 METs (min/day)	
Pedometer													
Aldhahir (2021)	Yamax Digi-Walker SW-200	Left side of waist	Yes (a diary card was provided for each period)	14 days	Not reported	Not reported	Not reported	Not reported	Not reported	Not reported	No	Step count (steps/day)	
Altenburg (2015)	Yamax Digi-Walker SW-200	Not reported	Yes (recorded number of steps per day in a diary)	2 weeks (mean of last week used for analysis)	Not reported	Not reported	5 days (during second week)	Minimum	Not reported	Not reported	No	Step count (steps/day)<br><br>Daily physical activity (n)	Daily physical activity (step count + metabolic equivalents [calculated from the activities recorded in diary using compendium of physical activities; Ainsworth (2000)])
de Blok (2006)	Yamax Digi-Walker SW-200	Waist	Yes (recorded number of steps per day in a diary)	7 days	Not reported	Not reported	Not reported	Not reported	Not reported	Not reported	No	Step count:<br><br>Steps/day (based on 7 days)<br><br>Steps/day (based on 4 days)<br><br>Steps/day	7 days (complete week)<br><br>4 days (days without rehab, including 1 weekend day)<br><br>6 days (days

												(based on 6 days)	without rehab+2 weekends)
Duiverman (2008)	Yamax Digi-Walker SW-200	Not reported	Yes (participants asked to record number of steps per day)	10 days	Not reported	Not reported	Not reported	Not reported	Not reported	Not reported	No	Step count (steps/day)	
Effing (2011)	Yamax Digi-Walker SW-200	Not reported	Not reported	7 days	Not reported	Not reported	Not reported	Not reported	Not reported	Not reported	No	Step count (steps/day)	
Felcar (2018)	Yamax Power-Walker-PW610	Not reported	Not reported	6 days	12 hours	Not reported	Not reported	Not reported	Not reported	Not reported	No	Step count (steps/day)<br><br>Estimated daily energy expenditure	
Nolan (2017) / Polgar (2021)	Yamax Digi-Walker CW700	Not reported	Yes: noted pedometer measured step count in trial diary	7 conse-cutive days	Not reported	Not reported	Not reported	Not reported	Not reported	Not reported	No	Step count (steps/day)	
Varas (2018)	OMRON Walking Style X Pocket HJ-320e	Waist next to right hip	Not reported	7 conse-cutive days	Not reported	Not reported	Not reported	Not reported	Not reported	Not reported	No	Step count (steps/day)	

*Reported in cited study.

Supplementary material P – Quality and reporting details of questionnaires for studies using questionnaire-based measures of movement behaviours

Author (Year)	Questionnaire/question	Recall period	Handling missing data
Armstrong (2021)	Clinical PROactive Physical Activity in COPD (C-PPAC)	7-day	Not reported
Bentley (2020)	Community Healthy Activities Model Programme for Seniors (CHAMPS)	4 weeks*	Not reported
Cameron-Tucker (2016)	Smoking, Nutrition, Alcohol consumption, Physical activity, Psychological well-being, and Symptom management (SNAPPS)	4 weeks	ITT (last case carried forward)
Gaunaurd (2014)	International Physical Activity Questionnaire (IPAQ)	7-day	Not reported
Kesten (2008)	Activities Questionnaire (created for the study)	2 weeks	Not reported
O'Neill (2018)	IPAQ	Not reported	Not reported (reasons for missing data reported)
Rausch Osthoff (2021)	IPAQ	7-day	Not reported
Troosters (2018)	Daily version of the C-PPAC (D-PPAC)	Daily	Not reported
Varas (2018)	Modified Baecke	Not reported	Not reported

*Reported in cited study.
